# Supplementary figures and images for: Protocol for a prospective double-blind, randomised, placebo-controlled feasibility trial of octreotide infusion during liver transplantation
Source: BMJ Open. 2021 Dec 2;11(12):e055864. doi: 10.1136/bmjopen-2021-055864 (PMC8640665; doi:10.1136/bmjopen-2021-055864)

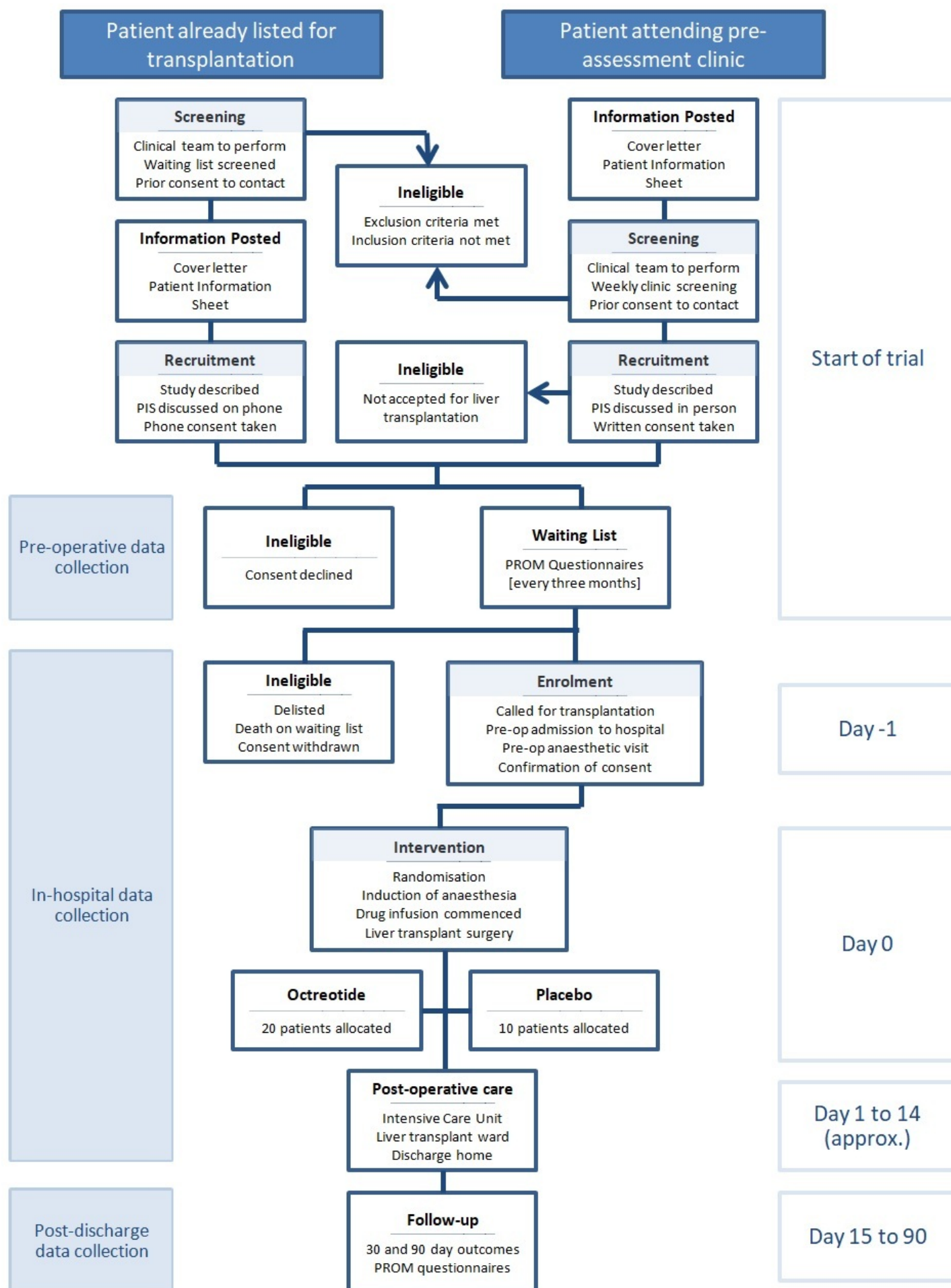

Supplement: Supplementary data [file bmjopen-2021-055864supp001.pdf]
