# Supplementary material for: Protocol for a prospective double-blind, randomised, placebo-controlled feasibility trial of octreotide infusion during liver transplantation
Source: BMJ Open. 2021 Dec 2;11(12):e055864. doi: 10.1136/bmjopen-2021-055864 (PMC8640665; doi:10.1136/bmjopen-2021-055864)
Supplement: Supplementary data [file bmjopen-2021-055864supp002.pdf]

Study Number: IRAS 278918
Sponsor Reference number 17/0508
Centre Number:
Patient Identification Number/Subject Number for this trial:

INFORMED CONSENT FORM (ICF)

In-Person Consent

Project Title Assessing the Impact of Octreotide Infusion during Liver Transplantation

Confirmation of consent to be performed on admission for transplantation
Points one to seven to be confirmed
Patient confirmation signature overleaf

Please initial box

1. I confirm that I have read and understand the information sheet dated 15.01.2021 (version 1.0) for the above study. I have had the opportunity to consider the information, ask questions and have had these answered satisfactorily. ☐

2. I understand that my participation is voluntary and that I am free to withdraw at any time without giving any reason, without my medical care or legal rights being affected. ☐

3. I understand that relevant sections of my medical notes and data collected during the study, may be looked at by individuals from the sponsor of the trial (University College London) and responsible persons authorised by the sponsor, from regulatory authorities or from the NHS Trust, where it is relevant to my taking part in this research. I give permission for these individuals to have access to my records. ☐

4. I agree to my GP being informed of my participation in the study. ☐

5. I consent to the collection, processing, reporting, storage and transfer within and outside Europe of my anonymised data for healthcare and/or medical research purposes in relation to this research. I understand that I will not be directly identifiable except to the study doctor and his/her study team. ☐

6. I agree to take part in the above study. ☐

7. After the entire study has completed I would like to receive a letter indicating which medication (octreotide or placebo) I received. [Optional]. ☐

Fabes J, *et al. BMJ Open* 2021; 11:e055864. doi: 10.1136/bmjopen-2021-055864

Name of patient | Date | Signature

Name of person taking consent | Date | Signature

Name of Chief Investigator | Date | Signature
(if different to the person taking consent)

When completed: 1 for participant; 1 (original) for researcher site file; 1 to be kept in medical notes. Please file a copy of the GP letter in the medical records and write a note in the medical record stating when the patient consented and date when the GP letter was sent.

Confirmation of consent on admission for transplantation

Name of patient | Date | Signature

Name of person taking consent | Date | Signature

Fabes J, *et al. BMJ Open* 2021; 11:e055864. doi: 10.1136/bmjopen-2021-055864
