# Supplementary material for: Protocol for a prospective double-blind, randomised, placebo-controlled feasibility trial of octreotide infusion during liver transplantation
Source: BMJ Open. 2021 Dec 2;11(12):e055864. doi: 10.1136/bmjopen-2021-055864 (PMC8640665; doi:10.1136/bmjopen-2021-055864)
Supplement: Supplementary data [file bmjopen-2021-055864supp003.pdf]

**Full title of trial**	A double-blind randomised placebo-controlled feasibility study to assess the impact of octreotide infusion during liver transplantation on post-operative renal failure.
**Short title**	A feasibility study of octreotide infusion during liver transplant.

Patient Information Sheet

Study title: A feasibility study of octreotide infusion during liver transplant.

Protocol Reference Number: Sponsor Ref number: 17/0508

We would like to invite you to take part in a research study. Before you decide we would like you to understand why the research is being done and what it would involve for you. One of our team will go through the information sheet with you when you attend hospital for your assessment and answer any questions you have. We would suggest this should take about ten minutes. Talk to others about the study if you wish.

Part 1 tells you the purpose of this study and what will happen to you if you take part. Part 2 gives you more detailed information about the conduct of the study.

Ask us if there is anything that is not clear. Take time to decide whether or not you wish to take part.

Part 1

What is the purpose of the study?

Liver transplant surgery can lead to effects on the kidneys requiring medical support including kidney dialysis. The surgery also has a risk of bleeding and the need for blood transfusion. These complications can slow down recovery after the operation and decrease the chances of the new liver working.

We are investigating a drug called octreotide that is very similar to a natural hormone present in the human body called somatostatin. Octreotide is already used regularly to treat kidney failure in liver disease. We already have evidence that octreotide improves how well the kidney creates urine during and after liver transplant surgery. There is also some evidence that octreotide improves patients' blood pressure in liver disease and during liver transplant surgery and this helps improve kidney function. One published study showed that octreotide might also reduce the amount of blood transfusion required during transplant surgery.

Fabes J, *et al. BMJ Open* 2021; 11:e055864. doi: 10.1136/bmjopen-2021-055864

We are running a research study to find out whether octreotide can improve kidney function during and after transplant surgery and leads to fewer patients needing to have kidney support. We will also look at whether octreotide helps stabilise blood pressure during surgery, reduce bleeding and the need for blood transfusion.

Octreotide is used regularly in some hospitals during liver transplant surgery but it is not clear whether it is beneficial. The quality of research that has been done on octreotide in liver transplant surgery is of poor quality. This research study aims to show if octreotide does have a benefit or not and whether this leads to long-term improvements in patients' health including their survival, the function of the new liver and quality of life.

Why have I been invited?

You have been invited to take part in this clinical trial because you have been referred to either the Royal Free or Queen Elizabeth Hospital for a liver transplant and meet all of the research study criteria for patients to be included. We foresee around thirty patients being involved in this research project over the next two years.

The research team running this clinical trial includes specialists from the liver transplant team, anaesthesia and intensive care as well as liver and kidney specialists.

Is there anything that would stop me from taking part?

If you are undergoing surgery to transplant any organs other than a liver transplant at the same time or if you have previously received any organ transplant, then you would not be able to take part in this study. You would also not be able to take part if you are receiving a 'living donor' liver organ, are suffering from severe liver failure called fulminant hepatic failure or if you have a known allergy to octreotide.

Do I have to take part?

It is up to you to decide to join the study. We will describe the study and go through this information sheet. If you agree to take part, we will then ask you to sign a consent form. You are free to withdraw at any time without giving a reason. Whether or not you are involved in this study, or if you decide to withdraw from the study at any time, will not affect the care you receive. Your involvement in this study will not have any effect on the chances of you receiving a liver transplant.

What will happen to me if I take part?

If you decide to take part then we will ask you to sign an informed written consent form prior to the study procedure.

All of the study takes place in the liver transplant operating theatre while you are under general anaesthetic at the Royal Free or Queen Elizabeth Hospital.

Fabes J, et al. BMJ Open 2021; 11:e055864. doi: 10.1136/bmjopen-2021-055864

Your experience of surgery and your care both before and after the transplant procedure will be identical to patients who are not taking part in the study.

In order to find out whether or not octreotide provides a benefit to patients we need to compare patient outcomes between those who have and have not had octreotide. To make the study as effective as possible we will compare the octreotide drug to a placebo. A placebo is a dummy treatment that looks like the real thing but is not. It contains no active ingredient. Patients who agree to be part of this study will receive either an infusion of octreotide or an infusion of an inactive drug (a saline solution).

To make the study as high a quality of research as is possible it will be performed in a 'double blind' fashion. In a 'blind trial' you will not know which treatment group you are in. As this trial is a 'double blind trial', neither you nor your doctor will know in which treatment group you are (although, if your doctor needs to find out he/she can do so).

Sometimes we don't know which way of treating patients is best. To find out, we need to compare different treatments. We put people into groups and give each group a different treatment. The results are compared to see if one is better. To try to make sure the groups are the same to start with, each patient is put into a group by chance (randomly). You have a two-in-three chance of your receiving the active drug (octreotide) in this study, and a one-in-three chance of the inactive drug.

This study is known as a 'feasibility study'. The idea of this type of research study is to find out whether a much larger trial would be possible and how it should be designed. The design of the study is identical to a larger trial but has a smaller number of patients in it.

Neither you nor the doctors looking after you will be aware of if you receive the active drug or not. We will monitor you very closely and record your data throughout your transplant and whilst on intensive care and the ward. The infusion will be started after you are asleep for your liver transplant and be stopped before you are transferred to the intensive care unit.

You will have received this Patient Information Sheet either by post or during your visit to The Royal Free London or Queen Elizabeth Hospital Birmingham. A member of the research team will contact you by phone if you are at home, or visit you whilst you are in the hospital to fully explain this study and offer you the opportunity to be involved. If you wish to take part we will complete a consent form with you when you attend the hospital on the day of your liver transplant.

Your involvement in the research project will be from the time you consent to being involved until ninety days after your liver transplant surgery as we will collect information during your recovery from the transplant procedure.

When you receive this Patient information leaflet, you will discuss the information with family, friends and/or GP. If interested then contact study team, who will discuss the study with you and you can register an expression of interest and if eligible you will be consented to d be involved in study. If you do not proceed to transplant over the study period you cannot be included in the trial.

The study will involve a small number of questionnaires but will not require you to attend any additional clinics or hospital appointments. These include:

- The completion of an written informed consent form,
- The completion of two quality of life questionnaires (taking roughly thirty minutes) during the pre-operative assessment,
- The completion of two quality of life questionnaires around three months after surgery. These will be done at your routine follow-up appointment and take roughly thirty minutes.

Patients who agree to take part in this study will receive all normal care and treatments for their liver transplant, no treatments will be withheld as part of this study. There is no video or audiotaping or photography involved in this study.

Expenses and payments

There are no expenses or payments involved in this study.

What are the possible disadvantages and risks of taking part?

Octreotide is an artificial version of a hormone that the body produces constantly to help regulate the gut and digestion. Octreotide is used routinely in the NHS for treatment of kidney failure and bleeding in liver disease. Some hospitals use octreotide routinely during liver transplant surgery and feel it provides benefits. Octreotide is a used quite commonly in patients who have liver disease. It is generally regarded as a very safe medication.

However, there are risks with all medical treatments. Effects that have been found from the use of octreotide are:

- Changes in blood sugar levels
- A slowing down in the heart rate
- Changes in heart rhythm

During and after liver transplant surgery the heart rate and rhythm is monitored constantly and if there are any concerns this can be treated. Your blood sugar levels are checked approximately hourly throughout surgery and at a similar frequency

Fabes J, *et al. BMJ Open* 2021; 11:e055864. doi: 10.1136/bmjopen-2021-055864

after surgery on the intensive care unit. If there are concerns regarding your blood sugar levels these are treated routinely.

If at any point during your surgery there is a concern that the medication is causing any problems that cannot be easily treated then the trial medication will be stopped.

There are no risks associated with the placebo infusion.

Before participating you should consider if this will affect any insurance you have and seek advice if necessary.

What are the effects of any treatment received when taking part?

Octreotide has been used in the NHS for a significant period of time and the effects of the medication are well understood.

Octreotide can cause low blood sugar levels in patients with diabetes and increase the effect of drugs taken to control blood sugar levels. Octreotide can also increase blood sugar levels in patients with and without diabetes. All patients in this study will have regular monitoring of blood sugar concentrations throughout their surgery and afterwards on the intensive care unit. All medicines affecting blood sugar levels are stopped routinely before surgery and blood sugar levels are controlled as part of routine care after surgery.

Octreotide may increase the effect of some medications that slow the heart or adjust the heart rhythm. During surgery and while on the intensive care unit the heart rate and rhythm of all patients is monitored continuously as part of routine care and kept in a safe range.

Octreotide can slow down how the body breaks down the medication codeine and this may reduce the pain relief provided by this medication. Codeine is not used as part of routine pain relief for patients who have undergone liver transplant surgery.

Octreotide may decrease the levels of a drug used to suppress the immune system called cyclosporine. Cyclosporin used to be used to reduce the change that the immune system would reject the new liver that had been transplanted. Cyclosporin is no longer used to suppress the immune system in liver transplantation.

There are no effects associated with the placebo infusion.

Anaesthetists, intensive care doctors, and other medical practitioners involved in the care of patients involved in this study will be aware of these possible effects of octreotide. Members of the research and pharmacy team who care for patients on this research study will monitor patients' medications to ensure the chance of effects is made as small as possible.

Fabes J, *et al. BMJ Open* 2021; 11:e055864. doi: 10.1136/bmjopen-2021-055864

We would expect the body to have removed all the octreotide medication within 48 hours after surgery. As such, there will be no concerns regarding octreotide after you have left hospital following your liver transplant surgery.

There are no recognized risks from octreotide to pregnant women. A pregnancy test is required for all women who are at are at risk of pregnancy before they can undergo liver transplant surgery. A positive pregnancy test would prevent you from taking part in this study. If you take part in this study there are no restrictions on you becoming pregnant following your liver transplant surgery, other than those given to you by your clinical care team.

What are the possible benefits of taking part?

We cannot promise the study will help you but the information we get from this study will help improve the treatment of people who are receiving future liver transplants.

Octreotide use during liver transplant surgery is a standard part of care in some of the world's top transplant institutes. The evidence we have so far suggests that octreotide infusions may reduce the chances of kidney failure after surgery, improve blood pressure, reduce bleeding and the need for blood transfusion. As such, if you receive the octreotide infusion your care may benefit, although we cannot be certain.

What happens when the research study stops?

Only a single treatment of octreotide or placebo will be given in this study. There is no evidence to suggest that further doses of octreotide after surgery would be of any benefit.

Your medical care after liver transplant surgery will be identical to all other patients who are not part of this research study. As such, there will be no changes after the research study stops.

It will not be possible to tell patients which medication they were given during the research study.

Once the study has been completed the data will be analysed and this may suggest that we should use Octreotide routinely. If so this could be introduced for future transplant patients. It is more likely that the data will indicate that a larger study is required to demonstrate whether Octreotide is beneficial in this situation.

What if there is a problem?

If you seem to have an adverse reaction to this medication, we will immediately stop the infusion of the trial medication and treat the complication if needed. We will document what has happened and inform you of what has happened.

Fabes J, *et al. BMJ Open* 2021; 11:e055864. doi: 10.1136/bmjopen-2021-055864

Any complaint about the way you have been dealt with during the clinical trial or any possible harm you might suffer will be addressed. The detailed information concerning this is given in Part 2 of this information sheet. If you have any concerns or complaints you should contact your study doctor in the first instance.

Will my taking part in the study be kept confidential?

Yes. We will follow ethical and legal practice and all information about you will be handled in confidence. The details are included in Part 2.

Fabes J, *et al. BMJ Open* 2021; 11:e055864. doi: 10.1136/bmjopen-2021-055864

Contact Details

Your Doctor

Queen Elizabeth Hospital, Birmingham

Consultant Anaesthetist

Royal Free Hospital, London

Consultant Anaesthetist and Intensivist

Your Research Nurse

Insert local contact details

Name

Tel. Number:

This completes Part 1 of the Information Sheet.

If the information in Part 1 has interested you and you are considering participation, please read the additional information in Part 2 before making any decision.

Part 2

What if relevant new information becomes available?

Sometimes we get new information about the treatment being studied. If this happens, we will tell you about it and discuss whether you want to or should continue in the study. If you decide not to carry on there will be no changes to your normal care before and after your transplant surgery. If you decide to continue in the study we will ask you to sign an updated consent form.

If the study is stopped for any other reason, we will tell you why and ensure your continuing care is unaffected.

What will happen if I don't want to carry on with the study?

You can withdraw from the study at any time without giving a reason, either before or after your liver transplant surgery. It will not affect your future care in any way.

If you choose to withdraw from the study before your liver transplant surgery then you will not be given the trial medication and any data that can be identified as yours can be destroyed if you wish.

If you choose to withdraw from the study after your liver transplant surgery then we would continue to collect and inspect data regarding drug safety and effects. We would also request that your data is used for the study analysis. However, we would not collect any other data and data not relating to this, for instance to do with your quality of life, would be destroyed if you wished. This is because we would have duty of care to you to ensure that you were safe having received the study medication.

What if there is a problem?

If you have a concern about any aspect of this study you should speak directly to the study organiser or a member of the research team. The anaesthetic and intensive care team involved in your care will be able to contact them for you directly. If you wish to complain about any aspect of the way in which you have been approached or treated during the course of this study, you can contact the study doctors through their contact details, below.

You may also wish to contact the Royal Free or Queen Elizabeth Hospital Patient Advice and Liaison Service on 020 7472 6446 (Royal Free) or 0121 424 0808 (Queen Elizabeth Hospital). They offer offers support, information and assistance to patients, relatives and visitors about medical services and hospital care.

Every care will be taken in the course of this clinical trial. However, in the unlikely event that you are injured by taking part, compensation may be available.

Fabes J, *et al. BMJ Open* 2021; 11:e055864. doi: 10.1136/bmjopen-2021-055864

If you suspect that the injury is the result of the Sponsor's (University College London) or the hospital's negligence, then you may be able to claim compensation. After discussing with your study doctor, please make the claim in writing to the Dr. Michael Spiro who is the Chief Investigator for the clinical trial and is based at The Royal Free Hospital, Department of Anaesthesia. The Chief Investigator will then pass the claim to the Sponsor's Insurers, via the Sponsor's office. You may have to bear the costs of the legal action initially, and you should consult a lawyer about this.

Participants may also be able to claim compensation for injury caused by participation in this clinical trial without the need to prove negligence on the part of University College London or another party. You should discuss this possibility with your study doctor in the same way as above.

Regardless of this, if you wish to complain, or have any concerns about any aspect of the way you have been approached or treated by members of staff or about any effects (adverse events) you may have experienced due to your participation in the clinical trial, the normal National Health Service complaints mechanisms are available to you. Please ask your study doctor if you would like more information on this. Details can also be obtained from the Department of Health website: http://www.dh.gov.uk.

Will my taking part in this study be kept confidential?

If you consent to take part in this study, the records obtained while you are in this study as well as related health records will remain strictly confidential at all times. The information will be held securely on paper and electronically at your treating hospital and the main hospital site managing this research (The Royal Free Hospital, London and University Hospitals Birmingham NHS Foundation Trust) under the provisions of the 1998 Data Protection Act. Your name will not be passed to anyone else out the research team or the Sponsor (UCL), who is not involved in the trial. You will be allocated a trial number, which will be used as a code to identify you on all trial forms. Any information about you which leaves the hospital/surgery will have your name and address removed so that you cannot be recognised.

Your records will be available to people authorised to work on the trial but may also need to be made available to people authorised by the Sponsor, which is the organisation responsible for ensuring that the study is carried out correctly. By signing the consent form you agree to this access for the current study and any further research that may be conducted in relation to it, even if you withdraw from the current study. Data collected during this phase of the study will be included in a larger follow-on study performed in an identical fashion.

The information collected about you may also be shown to authorised people from the UK Regulatory Authority (the Medicines and Healthcare Products Regulatory Authority); this is to ensure that the study is carried out to the highest possible scientific standards. All will have a duty of confidentiality to you as a research participant.

Fabes J, *et al. BMJ Open* 2021; 11:e055864. doi: 10.1136/bmjopen-2021-055864

If you withdraw consent from further study treatment, unless you object, your data and samples will remain on file and will be included in the final study analysis.

In line with the regulations, at the end of the study your data will be securely archived for a minimum of 20 years. Arrangements for confidential destruction will then be made.

Anonymised Data collected during the study may be transferred for the purpose of analysis to associated researchers within/out the European Economic Area. Some countries outside of Europe may not have laws which protect your privacy to the same extent as the Data Protection Act in the UK or European Law. The Sponsor of the trial will take all reasonable steps to protect your privacy.

Will my GP be informed of my involvement?

With your permission, your GP, and other doctors who may be treating you, will be notified that you are taking part in this study.

What will happen to any samples I give?

No samples will be taken.

Will any genetic tests be done?

No genetic tests will be performed.

What will happen to the results of the research study?

The results of the study will be available after it finishes and will usually be published in a medical journal or be presented at a scientific conference. The data will be anonymous and none of the patients involved in the trial will be identified in any report or publication.

Should you wish to see the results, or the publication, please ask your study doctor. We will send all interested patients a copy of the study results and will arrange a presentation of the research findings to the participants and the wider liver transplant community.

Who is organising and funding the research?

The research is being funded by the National Institute for Health Research as part of their Research for Patient Benefit programme. The doctors involved in setting up and running this research project are not being paid to do so. The doctors and wider research team do not have any conflict of interests in this study and no companies or members of the pharmaceutical industry are involved in any way.

Who has reviewed the study?

Fabes J, *et al. BMJ Open* 2021; 11:e055864. doi: 10.1136/bmjopen-2021-055864

All research in the NHS is looked at by independent group of people, called a Research Ethics Committee, to protect your interests. This study has been reviewed and given favourable opinion by East Midlands - Leicester South Research Ethics Committee.

Further information and contact details

You are encouraged to ask any questions you wish, before, during or after your treatment. If you have any questions about the study, please speak to your study nurse or doctor, who will be able to provide you with up to date information about the drug(s)/procedure(s) involved. If you wish to read the research on which this study is based, please ask your study nurse or doctor. If you require any further information or have any concerns while taking part in the study, please contact one of the following people:

Your Doctor

Queen Elizabeth Hospital, Birmingham

Consultant Anaesthetist

Royal Free Hospital, London

Consultant Anaesthetist and Intensivist

Your Research Nurse

Name

Tel. Number:

Alternatively, if you or your relatives have any questions about this study you may wish to contact one of the following organisations that are independent of the hospital at which you are being treated:

If you decide you would like to take part then please read and sign the consent form. You will be given a copy of this information sheet and the consent form to keep. A copy of the consent form will be filed in your patient notes, one will be filed with the study records and one may be sent to the Research Sponsor.

You can have more time to think this over if you are at all unsure.

Fabes J, *et al. BMJ Open* 2021; 11:e055864. doi: 10.1136/bmjopen-2021-055864

Thank you for taking the time to read this information sheet and to consider this study.

Fabes J, *et al. BMJ Open* 2021; 11:e055864. doi: 10.1136/bmjopen-2021-055864
