# Supplementary material for: Protocol for a prospective double-blind, randomised, placebo-controlled feasibility trial of octreotide infusion during liver transplantation
Source: BMJ Open. 2021 Dec 2;11(12):e055864. doi: 10.1136/bmjopen-2021-055864 (PMC8640665; doi:10.1136/bmjopen-2021-055864)
Supplement: Supplementary data [file bmjopen-2021-055864supp004.pdf]

Appendix 4: TMG and TSC terms of reference and functions

Responsibilities of the combined Trial Steering Committee and Data Monitoring Committee

To act as the oversight body for the trial on behalf of the Sponsor and Funder.

The role of the TSC is to provide oversight for the trial and provide advice through its independent Chair to the Chief Investigator, the TMG, trial Sponsor, Funder, and host institution on all aspects of the trial. The rights, safety and well-being of the trial participants are the most important consideration and should prevail over the interests of science and society.

Functions of the TSC include:

- Provide expert oversight of the trial
- Maintain confidentiality of all trial information not already in the public domain
- Review regular progress reports from the trial team
- Monitor recruitment rates and review strategies proposed to deal with any recruitment problems
- Make decisions regarding continuation or termination of the trial
- Assess the impact and relevance of any accumulating external evidence
- Monitor quality of the data and review strategies proposed to improve data collection
- Monitor follow-up rates and review strategies proposed to deal with problems
- Monitor sites that are deviating from the protocol
- Review any proposed amendments to the protocol
- Review proposals by the TMG concerning any change to the design of the trial, including additional sub-studies
- Oversee the timely reporting to trial results
- Review the statistical analysis plan
- Review the publication policy
- Review the main trial manuscript
- Review any abstracts and presentations of results during the trial
- Approve external or early internal requests for release of data, subsets of data or samples including clinical data.

Responsibilities of the Trial Management Group

- Input into and comment on the protocol and data collection methods
- Promote the trial
- Develop strategies to encourage recruitment and address any issues with recruitment at participating sites
- Be involved in the day-to-day running of the trial by supporting the CI and trial coordinators
- Provide clinical or other expert guidance to the trial coordinators and participating sites on trial- based matters such as clinical and practical queries and interpretation of information recorded on eCRFs or other data collection tools
- Maintain confidentiality of any trial information that is not in the public domain
- Respond to trial correspondence and any questions in a timely fashion
- Review data collection at sites
- Input into the monitoring and classification of SAEs where clinically appropriate
- Input into the meetings of the TSC, if appropriate
- Provide responses to any issues or concerns raised by TSC
- Be aware of accumulating external evidence and assess its impact and relevance
- Input into the development of the Statistical Analysis Plan where appropriate
- Input into the interpretation and trial report writing
- Report to the TSC regarding trial progress
