# Supplementary material for: Protocol for a prospective double-blind, randomised, placebo-controlled feasibility trial of octreotide infusion during liver transplantation
Source: BMJ Open. 2021 Dec 2;11(12):e055864. doi: 10.1136/bmjopen-2021-055864 (PMC8640665; doi:10.1136/bmjopen-2021-055864)
Supplement: Author's manuscript [file bmjopen-2021-055864.draft_revisions.pdf]

BMJ Open

BMJ Open is committed to open peer review. As part of this commitment we make the peer review history of every article we publish publicly available.

When an article is published we post the peer reviewers' comments and the authors' responses online. We also post the versions of the paper that were used during peer review. These are the versions that the peer review comments apply to.

The versions of the paper that follow are the versions that were submitted during the peer review process. They are not the versions of record or the final published versions. They should not be cited or distributed as the published version of this manuscript.

BMJ Open is an open access journal and the full, final, typeset and author-corrected version of record of the manuscript is available on our site with no access controls, subscription charges or pay-per-view fees (http://bmjopen.bmj.com).

If you have any questions on BMJ Open's open peer review process please email info.bmjopen@bmj.com

BMJ Open

Protocol for a prospective double-blind, randomised, placebo-controlled feasibility trial of octreotide infusion during liver transplantation

Journal:	*BMJ Open*
Manuscript ID	bmjopen-2021-055864
Article Type:	Protocol
Date Submitted by the Author:	26-Jul-2021
Complete List of Authors:	Fabes, Jez; University of Plymouth, Peninsula Medical School; Royal Free London NHS Foundation Trust, Department of Anaesthesia<br>Ambler, Gareth; University College London, Department of Statistical Science<br>Shah, Bina; University College London Division of Surgery & Interventional Science<br>Williams, Norman; University College London, UCL Division of Surgery & Interventional Science<br>Martin, Daniel; University of Plymouth, Peninsula Medical School; University College London, Division of Surgery<br>Davidson, Brian; University College London, Division of Surgery<br>Spiro, Michael; University College London, Division of Surgery
Keywords:	Adult anaesthesia < ANAESTHETICS, Hepatobiliary disease < GASTROENTEROLOGY, Adult intensive & critical care < INTENSIVE & CRITICAL CARE, Hepatology < INTERNAL MEDICINE, Hepatobiliary surgery < SURGERY, Transplant surgery < SURGERY

SCHOLARONE™
Manuscripts

I, the Submitting Author has the right to grant and does grant on behalf of all authors of the Work (as defined in the below author licence), an exclusive licence and/or a non-exclusive licence for contributions from authors who are: i) UK Crown employees; ii) where BMJ has agreed a CC-BY licence shall apply, and/or iii) in accordance with the terms applicable for US Federal Government officers or employees acting as part of their official duties; on a worldwide, perpetual, irrevocable, royalty-free basis to BMJ Publishing Group Ltd ("BMJ") its licensees and where the relevant Journal is co-owned by BMJ to the co-owners of the Journal, to publish the Work in this journal and any other BMJ products and to exploit all rights, as set out in our licence.

The Submitting Author accepts and understands that any supply made under these terms is made by BMJ to the Submitting Author unless you are acting as an employee on behalf of your employer or a postgraduate student of an affiliated institution which is paying any applicable article publishing charge ("APC") for Open Access articles. Where the Submitting Author wishes to make the Work available on an Open Access basis (and intends to pay the relevant APC), the terms of reuse of such Open Access shall be governed by a Creative Commons licence – details of these licences and which Creative Commons licence will apply to this Work are set out in our licence referred to above.

Other than as permitted in any relevant BMJ Author's Self Archiving Policies, I confirm this Work has not been accepted for publication elsewhere, is not being considered for publication elsewhere and does not duplicate material already published. I confirm all authors consent to publication of this Work and authorise the granting of this licence.

For peer review only

Title

Protocol for a prospective double-blind, randomised, placebo-controlled feasibility trial of octreotide infusion during liver transplantation

J. Fabes[1], G. Ambler[2], B. Shah[2], N. Williams[2], D. Martin[1], B. Davidson[3], M. Spiro[3]

1 University

For peer review only

Abstract

Introduction

Liver transplantation is a complex operation that can provide significant improvements in quality of life and survival to the recipients. However, serious complications are common and include major haemorrhage, hypotension and renal failure. Blood transfusion and the development of acute kidney injury lead to both short- and long-term poor patient outcomes, including an increased risk of death, graft failure, length of stay and reduced quality of life. Octreotide may reduce the incidence of renal dysfunction, perioperative haemorrhage and enhance intraoperative blood pressure. However, octreotide does have risks, including resistant bradycardia, hyper- and hypoglycaemia and QT prolongation. Hence a randomised controlled trial (RCT) of octreotide during liver transplantation is needed to determine the cost efficacy and safety of its use; this study represents a feasibility study prior to this RCT.

Methods and analysis

We describe a multi-centre, double-blind, randomised, placebo-controlled feasibility study of continuous infusion of octreotide during liver transplantation surgery. We will recruit 30 adult patients at two liver transplant centres. A blinded infusion during surgery will be administered in a 2:1 ratio of octreotide:placebo. The primary outcomes for this study are designed to determine the feasibility of this study design. Secondary outcome measures based on efficacy and safety will help shape future trials by assessing potential primary outcome measures and monitoring safety end-points. No formal statistical tests are planned. This manuscript represents study protocol number 1.3, dated 2nd June 2021.

Ethics and dissemination

This study has been given Research Ethics Committee approval. The main study outcomes will be submitted to an open-access journal.

Registration

The study is registered with ClinicalTrials.gov (NCT04941911) with recruitment due to start in August 2021 with anticipated completion in July 2022. WHO Trial Registration Data Set in Table 1.

Declaration of interests

The chief and principal investigators declare that they have no financial or other competing interests in this study.

Funding

This project is funded by the National Institute for Health Research (NIHR) under its Research for Patient Benefit (RfPB) Programme (Grant Reference Number PB-PG-0817-20023). The views expressed are those of the author(s) and not necessarily those of the NIHR or the Department of Health and Social Care.

Corresponding author

For peer review only

Dr. Jez Fabes, jeremy.fabes@nhs.net

Trial Sponsor

The Joint Research Office, University College London, United Kingdom

Neither the Sponsor nor the Funder have any role in study design, collection, management, analysis, and interpretation of data, writing of the study report or the decision to submit the report for publication.

Clinical trials unit

Surgical and Interventional Group, Division of Surgery & Interventional Science, University College London

Key words

Liver transplantation; octreotide; renal failure; patient reported outcome measures; feasibility.

Strengths and limitations of this study

- This study is prospective, randomised and designed to minimise the risk of bias throughout
- We have prioritised patient input throughout the development and design of this study
- A small sample size has been selected to demonstrate feasibility end points as a precursor to a larger efficacy and safety trial
- The secondary end points will provide clinically relevant data on which to design this cost effectiveness study

INTRODUCTION

Liver transplantation is a potentially life transforming intervention with excellent outcomes (one and five year survival of 94% and 84%, respectively[1]). It is, however, a major operation in patients with multiple co-morbidities and is associated with a 3% perioperative mortality rate and 70% complication rate. Intra-operative haemorrhage is serious and commonly occurs during mobilisation of the diseased liver, exacerbated by coagulopathy and abnormal vasculature due to portal hypertension. Bleeding can cause hypotension, reduced perfusion of the transplanted graft and increased incidence of acute kidney injury (AKI). Blood loss and transfusion are associated with increased morbidity, mortality and cost of transplantation and can have a long term detrimental effect on patient wellbeing and quality of life[2-4].

Octreotide is a synthetic analogue of somatostatin with an excellent safety profile[5] that may improve renal outcomes, reduce perioperative bleeding and increase exogenous vasopressor sensitivity during liver transplantation. Acute kidney injury following liver transplantation occurs in 25-60% of patients with normal renal function prior to surgery, with half of these patients requiring renal replacement therapy (RRT) after surgery[6, 7]. The need for post-transplant RRT increases the length of hospital stay and the risk of early allograft dysfunction, chronic kidney disease (CKD) and mortality[8, 9]. Risk factors for renal dysfunction following transplantation include intraoperative hypotension, lower quality donor grafts, hepatic ischemia-reperfusion injury, blood transfusion and pre-operative renal disease[10, 11]. Octreotide has been shown to enhance renal blood flow, glomerular filtration and urine output in decompensated liver cirrhosis[12], hepatorenal syndrome[13] and may also reduce the incidence of post-transplant renal injury[14, 15].

Major haemorrhage and transfusion during and after liver transplantation reduce patient and graft survival as well as increasing length of stay and the need for RRT[2-4, 16-18]. Haemorrhage risk during liver transplantation is multifactorial, including cirrhotic coagulopathy, portal hypertension and hyperfibrinolysis. Bleeding is driven by the presence of wide-spread dilated collaterals and a hyperdynamic splanchnic circulation. Octreotide causes splanchnic vasoconstriction thereby reducing splanchnic blood flow, collateralisation and portal venous pressures which may reduce surgical blood loss[19]. This correction of the abnormal blood distribution and venous pooling increases systemic blood pressure[20] and, when combined with a vasopressor, correlates with improved renal function[21]. Furthermore, increased systemic pressures and vasopressor sensitivity help preserve renal function and reduce myocardial injury[10, 22, 23].

Previous clinical studies of octreotide in liver transplantation

A retrospective cohort study of octreotide infusion during liver transplantation demonstrated a reduction in transfusion requirement by 2.3 units of packed red blood cells but this difference was not statistically significant[24]. A randomised controlled trial (RCT) of octreotide infusion during liver transplantation found an enhancement of noradrenaline effect on blood pressure with a mean increase of 8mmHg[15].

Risks

Octreotide is not currently licensed for use in liver transplantation. Common risks of octreotide include changes in blood sugar levels, heart rate and rhythm. The side effect profile of octreotide

For peer review only

during liver transplantation is unknown. The studies to date did not compare safety or adverse events between the octreotide and placebo arms[15], have not been fully published[25] or did not assess adverse events[24].

Rationale for this study

The background literature for the use of octreotide in liver transplantation is inconclusive and the current use in this setting is clinician and institution dependent. While there is reasonable evidence to support its use in this setting, octreotide has harmful effects which need to be considered against its efficacy and cost. Furthermore, patients may be unwilling to have non-essential medication and clinicians may not support its use in all transplant settings. In addition to determining the feasibility of a multi-site drug trial in liver transplantation, secondary end points will provide clinically relevant data on which to design a subsequent cost-effectiveness study.

Hypothesis

It is feasible to recruit and retain patients in a randomised study of continuous infusion of octreotide during liver transplantation surgery.

METHODS AND ANALYSIS

Trial design

The purpose of this study is to determine the feasibility of recruiting patients to a double-blind, randomised, placebo-controlled trial of intravenous octreotide during liver transplantation. We will recruit 30 adult patients at two liver transplant centres; The Royal Free London NHS Foundation Trust (Royal Free Hospital, RFH), and University Hospitals Birmingham NHS Foundation Trust (University Hospital Birmingham, UHB). A blinded infusion will be administered during transplantation surgery. Patients will be allocated in a 2:1 ratio to octreotide or placebo. All members of the research, anaesthetic, surgical, intensive care and nursing teams will be blinded to patient allocation; this includes all outcome assessors and data analysts. Patients will be blinded to their allocation and will have the option to find out which intervention they received after the study is complete. Unblinding will be carried out once data cleaning and analysis has been completed.

This protocol conforms to the Standard Protocol Items: Recommendations for Interventional Trials (SPIRIT)[26]. The RCT will be reported in line with the Consolidated Standards of Reporting Trials (CONSORT) statement[27]. This trial is non-commercial and does not intend to generate data for drug licensing. The study is registered with ClinicalTrials.gov (NCT04941911) with recruitment due to start in August 2021 with anticipated completion in July 2022.

Eligibility

Inclusion criteria

1. Adults aged 18 years and over accepted for liver transplant and on the transplant waiting list.
2. Undergoing liver transplantation.
3. Recipient of a whole or partial liver graft from a cardiac or brain dead donor.

For peer review only

Exclusion criteria

1. Lacking capacity to provide consent (according to the Mental Capacity Act, 2005).
2. Recipients of multiple solid organ transplants.
3. Previous receipt of a solid organ transplant.
4. Patients with acute or fulminant hepatic failure.
5. Patients receiving a live-donor liver graft.
6. Patients admitted to an intensive care unit (ICU) at the time of transplantation.
7. Preoperative requirement for RRT, including dialysis.
8. Known allergy or adverse reaction to octreotide.
9. Preoperative decision to use intraoperative RRT.
10. Patients enrolled in another interventional study that affects patient care during transplantation surgery or would be expected to have an effect on the study outcomes will be excluded from this study.

Patients who are enrolled in a study examining the impact of extracorporeal machine perfusion of the donated liver prior to transplantation will be eligible for this study.

Patient identification and recruitment

Participant screening and registration will be undertaken locally at each study site. Overall trial flow is outlined in Appendix 1.

Patients will be identified and recruited in two ways, depending on whether they are undergoing assessment for suitability for liver transplantation or have already been accepted onto the waiting list for transplantation. Screening will be performed by the clinical team, as will primary contact regarding engagement with research, prior to research team contact.

i. Patients undergoing assessment for liver transplantation will be sent the patient information sheet (PIS) for this study. Patients that meet the inclusion criteria will be approached during their in-patient assessment process to explain the study and for preliminary informed consent.

ii. Patients already listed for liver transplantation who meet the inclusion criteria will be sent the PIS. These patients will be contacted by phone, the study and PIS explained, and preliminary informed consent taken.

Due to the waiting time for transplant and the fixed duration of this study many patients who would be willing to enter the study will not be able to participate because a donor organ does not become available. Both groups of patients will therefore have their consent confirmed on the day of admission for surgery at which point, once the transplant surgery has been confirmed, the patient will be considered to be enrolled.

Consent

Informed consent will be obtained by a member of the research team and confirmed when the patient is admitted for surgery. The PIS and consent form (Appendices 3 and 4) will be reviewed and updated, if necessary, throughout the trial (e.g. where new safety information becomes available). Consent will be sought for use of participant data in further studies in relation to this research.

For peer review only

Study interventions

Patients will follow the standard protocol for liver transplantation at each study centre with the only study intervention being the provision of octreotide or placebo infusion. The study infusion will commence after induction of anaesthesia and prior to surgical incision. There will be no cross-over or dose escalation. The study infusate will be administered intravenously through an electronic infusion pump until completion of 50 ml of study medication or transfer to ICU, whichever is sooner. The maximum total dose of octreotide infused will be 1 mg, including an initial slow bolus followed by an infusion. Similarly, the maximum dose of 0.9% saline placebo administrated will be 50 ml. Where the infusion is completed prior to the end of surgery then no further study drug or placebo will be given.

Intervention group

Syringes will contain 50ml of octreotide acetate at 20mcg/ml in 0.9% w/v sodium chloride in water.

The octreotide infusion will commence with a 100 mcg bolus (5 ml) over 30 minutes with a subsequent infusion of 100 mcg (5 ml) per hour during surgery.

Comparator group

Syringes will contain 50ml of 0.9% w/v sodium chloride in water.

The control infusion will commence with a 5 ml bolus over 30 minutes with a subsequent infusion of 5 ml per hour during surgery.

Study drug

Octreotide is licensed in the UK and is being used outside of its marketed authorisation for the purpose of this trial. The Summary of Product Characteristics will be used as a safety reference and the dose will not exceed the maximum dose of 1.5 mg per day. A suitable marketed product will be sourced by the RFH Pharmacy Manufacturing Unit that allows seven days stability after reconstitution. The study drug will be prepared and released by a Qualified Person for use in the study by the holder of a Manufacturing and Import Authorisation (Investigational Medicinal Products) licence.

Overdose

Deviations from the treatment schedule or inadvertent administration of additional octreotide may lead to overdose. These will be reported in the case report form (CRF), the patient will be notified and the information and implications reported to the Sponsor. The patient would continue to be included in the trial. Where a serious adverse event (SAE) occurs in association with an overdose then the nature, dose and cause of the overdose will be included in an SAE report form. Participants who receive an overdose of octreotide will be followed up and treated in the usual manner for the trial.

Relevant symptoms of overdose include arrhythmia, hypotension, cardiac arrest, pancreatitis, hepatic steatosis, diarrhoea and lactic acidosis. Management is supportive.

For peer review only

Randomisation

Consented patients who proceed to transplantation will be randomised. Remote-site computerised random allocation will be performed with a centrally generated code (www.sealedenvelope.com) that will correlate with a coded syringe held in the secure study drug storage facility at each study centre. Patients will be stratified within the randomisation process by source of liver graft (brain death, DBD, or cardiac death donor, DCD) to ensure that patients are balanced for these factors across the two treatment groups. Patients will be randomly allocated to either the octreotide or placebo study groups in a 2:1 ratio. The randomisation list will be held centrally at a dedicated and independent off-site facility that will be contactable 24 hours a day.

Allocation concealment

The syringes of octreotide 20 mcg/ml (intervention) and sodium chloride 0.9% w/v (control) will appear identical and only identifiable by their trial-allocated code. No members of the research or clinical team will be aware of patient allocation or syringe contents. The allocation code will only be broken following completion of patient follow up, data cleaning and analysis; unless an emergency break code is requested. The study statistician will be provided only with information on treatment A or B.

Unblinding

Where required for medical or safety reasons a trial break code can be obtained from a nominated member of the research team who will remain blinded to patient allocation. Information on the indication and outcome of the unblinding will be forwarded to the Trial Steering Committee (TSC) and included in the final trial report. While Suspected Unexpected Serious Adverse Reaction (SUSAR) reports will include the treatment allocation, this information will not be available to the trial team and blinding will be maintained.

Outcomes

Primary outcome

The primary outcomes for this study are based on determining the feasibility of conducting a randomised controlled trial of octreotide versus placebo infusion during liver transplantation. The feasibility will be assessed by;

- Recruitment ratio (percentage of eligible patients undergoing transplantation who provide consent, target: ≥ 30%),
- Percentage of eligible patients who receive the study drug infusion in a blinded manner (target: ≥ 80% of patients),
- Incidence of drug-related serious unexpected adverse events (target: ≤ 20%),
- Completion of follow-up data collection (target: ≥ 90% patients),
- Patient refusal to enrol in study on admission for transplantation (target: ≤ 15%),
- Clinician refusal for inclusion or randomisation following patient recruitment (target: ≤ 15%).

Secondary outcomes

For peer review only

A number of secondary outcome measures will be collected to help shape future trials in terms of assessing potential primary outcome measures and monitoring safety end-points.

- Intra-operative cell salvage volume (as a proxy for blood loss),
- Intra-operative urine output during each surgical phase,
- Mean arterial blood pressure during each surgical phase,
- Requirement for and mean vasopressor and/or inotrope infusion rate during each surgical phase,
- Volume or mass of *de novo* packed red blood cells and other clotting products during surgery and on the ICU (within first 24 hours, 72 hours and one week),
- Incidence of AKI as defined by the Acute Kidney Injury Network stage 1 criteria (a 50% increase in serum creatinine from baseline or less than 0.5 ml/kg/hr urine output for 6-12 hours post-transplant) within 24 hours, 72 hours, one week,
- Incidence of postoperative RRT at 24 hours, 72 hours, one and two weeks postoperatively,
- Incidence of new or worsened CKD at thirty and ninety days,
- Mortality, early allograft dysfunction, graft loss and liver graft function at thirty and ninety days,
- Patient recorded outcome measures (PROM) preoperatively and at ninety days postoperatively;
    - Liver Disease Quality of Life questionnaire[28],
    - EuroQOL-5D-5L questionnaire[29].

Major safety outcomes will include;

- Unexpected or resistant bradycardia,
- Abnormal QTc interval (460ms in men, 470ms in women) or associated ventricular arrhythmia or Torsades de Pointes,
- Unexpected or resistant hypoglycaemia (blood sugar < 4 mmol/L),
- Potential allergic or anaphylactic reaction to any medication,
- Any report of an abnormal response to codeine or morphine,
- Development of venous or arterial thrombosis,
- Cardiac event including acute coronary syndrome, new heart failure, arrhythmia or resuscitated cardiac arrest.

Participant withdrawal

A participant may be withdrawn from the trial by their clinician whenever continued participation is considered to no longer be in the participant's best interests, but the reasons for doing so must be recorded. In these cases routine follow-up and safety data will be collected but not any further study-specific data.

A participant may withdraw from the trial at any point without impact on their ongoing care. No further data would be collected. However, data up to that point will be retained. Permission will be sought from patients wishing to withdraw to allow use of their routine follow-up data for trial purposes, especially safety data.

For peer review only

Sample size

Thirty patients will be recruited from two sites, with 20 in the study drug (octreotide) arm and 10 in the placebo. This sample will allow us to demonstrate the feasibility of conducting a full-scale randomised trial. This study is not powered to detect differences in treatment effect but will obtain estimates of an effect and its variance[30]. This sample size will allow us to estimate patient consent rate with a 95% confidence interval of ± 10% (based on 100 participants approached). The secondary outcome data will provide the required information for a sample size calculation for the primary end point of a subsequent trial[31]. Survival to discharge is above 96% for both study sites and hence it is likely that the ninety day follow data collection will be achieved for the majority of patients.

Recruitment rate

Over 275 patients per year will be eligible for study inclusion across the two study sites. With a ten month recruitment period, this requires a 13% recruitment rate. A recent interventional trial in liver transplantation at the RFH achieved a 53% recruitment rate[32]. Staff involvement and recruitment strategies will be similar in this study indicating this is a deliverable recruitment target.

Data collection

Data will be collected on trial-specific electronic case report forms (eCRFs) and anonymised data collated on a secure central trial database (MACRO, Elsevier, Amsterdam, Netherlands) on a server in UCL. Physical trial material will be stored locally at each study site in a secure setting. A trial specific data management standard operating procedure will contain details of the software to be used for the database, the process of database design, data entry, data quality checks, data queries, data security and database lock. At the end of the trial, all essential documentation will be archived securely for a minimum of 20 years from the declaration of the end of the trial.

All in-hospital data required for the study is part of routine medical care and as such is expected to have a high rate of completeness. Out-of-hospital data is similarly part of routine medical care. The completeness of study-specific PROM questionnaires performed before and after transplantation will be maximised through reminders by telephone and during clinic appointments.

Data will be analysed for completeness and accuracy through a check of 10% of patient records against the source data.

Third party data access will be assessed on a case-by-case basis by the Trial Management Group, approved by the Trial Steering Committee and limited specific anonymised data will be released, as appropriate. Patients will be consented for further analysis of anonymised data.

Statistical methods

A Statistical Analysis Plan will be approved by the Trial Steering Committee, prior to data analysis.

The following variables will be used to assess baseline comparability of the randomised groups;

- Study centre at which transplant performed,
- Age,
- Sex,

For peer review only

- The model for end-stage liver disease (MELD) score[33],
- The United Kingdom end-stage liver disease (UKELD) score[34],
- Aetiology of liver disease,
- The UK donor risk index[35],
- Organ graft undergoing normothermic machine perfusion,
- Presence of portal hypertension,
- Severity of pre-operative renal dysfunction (estimated glomerular filtration rate, eGFR),
- American Society of Anesthesiologists score,
- Pre-operative haemoglobin level,
- EuroQol-5D-5L questionnaire scores,
- Liver disease Quality of Life questionnaire score.

Patients will be analysed according to the groups to which there were randomised.

Primary outcomes

The primary outcome feasibility measures will be quantified using numbers (%). The degree of precision will be quantified using exact 95% confidence intervals.

Secondary outcomes

Numerical outcomes will be summarised using either mean (standard deviation) or medians (interquartile range) as appropriate. Binary and ordinal outcomes will be summarised using number (%). The degree of precision will be quantified using 95% confidence intervals. Differences between groups will be quantified using differences in means or proportions, as appropriate with corresponding 95% confidence intervals.

Since this is a feasibility study, no formal statistical tests are planned.

Sensitivity and other planned analyses

No sensitivity analyses are planned. There will be no interim analysis.

We will perform exploratory subgroup analyses for donor organ type (DCD and DBD), the presence of chronic kidney disease and known portal thrombosis (or if diagnosed at transplantation). We will also assess the impact of surgical approach (cross-clamp vs. any other approach), the use of machine perfusion vs. cold storage and hospital site.

Patient replacement

Patients who are randomised and allocated to a treatment arm but subsequently do not complete the trial will not be replaced.

Monitoring

Oversight committees

A Trial Management Group (TMG) and Trial Steering Committee (TSC) have been formed. The terms of reference and functions of these committees are provided in Appendix 2.

For peer review only

The TMG will include the Chief Investigators, co-investigators, trial staff, patient expert and study site Principle Investigators. The TMG will be responsible for overseeing the trial. The TMG will review recruitment figures, serious adverse events and substantial amendments to the protocol prior to submission to the Research Ethics Committee (REC).

Data monitoring

The TSC will represent a combined TSC and Data Monitoring Committee with expertise in performing and reporting clinical trials, statistical methods and clinical expertise for evaluation of post-transplant complications. The role of the TSC is to provide oversight for the trial and provide advice through its independent Chair to the Chief Investigator, the TMG, trial Sponsor, Funder, and host institutions on all aspects of the trial. The TSC will recommend any appropriate amendments/actions for the trial as necessary. The TSC is independent of the TMG, Funder and Sponsor and members have declared no competing interests.

Early stopping guidelines

The trial may be stopped before completion either on the recommendation of the TSC or the Sponsor and Chief Investigator. There will be no statistical stopping rules.

Adverse events

All adverse events will be recorded in the medical records in the first instance. All serious adverse events (SAEs) within 90 days of patient enrolment will be recorded in the eCRF with clinical symptoms, a brief description and date of the event. SAEs will be reported to the Sponsor within 24 hours. Where the event is unexpected and thought to be related to the intervention, this will be reported to the Health Research Authority within 15 days. Participants suffering an SAE will be followed up until clinical recovery is complete and laboratory results have returned to expected values, or until the event has stabilised. Follow-up will continue after completion of trial follow-up if necessary. For the purpose of analysis, SAEs prior to discharge, within 30 days and within 90 days of enrolment will be analysed and published.

The sponsor will notify the REC and MHRA of all suspected unexpected serious adverse reactions (SUSARs) within 15 days. SUSARs that are fatal or life-threatening will be notified to the MHRA and REC within 7 days of the sponsor learning of them.

Auditing

Given the short duration of this study no formal audit is planned. All documentation will be collected and recorded in keeping with Good Clinical Practice guidelines and will be available for external review or auditing if required.

ETHICS AND DISSEMINATION

Research ethics approval

This study has been given approval by the East Midlands - Leicester South Research Ethics Committee (IRAS reference: 278918, REC reference: 21/EM/0076) and the Health Research Authority. This feasibility study does not require MHRA approval.

For peer review only

Protocol amendments

Substantial protocol amendments will be reviewed by the TMG, TSC, and Sponsor prior to REC and Health Research Authority submission.

Any changes from this protocol paper will be highlighted in the study outcome publication.

Confidentiality

All investigators and trial site staff will comply with the requirements of General Data Protection Regulation with regard to the collection, storage, processing and disclosure of personal information and will uphold the Data Protection Act's core principles. All patient records will remain strictly confidential at all times. All patients recruited to the study will be allocated a unique trial identification number that will be used on all study-specific correspondence. All study information will be held securely on paper and electronically on eCRFs. Any patient information which leaves the hospital will only contain the trial identifier. Patient identifiable details will not be passed to anyone outside the research team or the study Sponsor.

Declaration of interests

The Chief and Principal Investigators and members of the TMG have declared that they have no financial or other competing interests.

Ancillary and post-trial care

The Sponsor holds insurance against claims from participants for injury caused by their participation in the trial. Each study site hospital will provide negligence insurance cover for harm caused by their employees. All ongoing care for the treatment of adverse events will be provided by the recruiting hospital.

Dissemination policy

A publication policy will be written and agreed by the TMG prior to trial data analysis. This will be agreed with the TSC. The main study outcomes will be submitted to an open-access journal.

Patient and public involvement

Interviews and engagement with liver transplantation patients and their families has been a key motivator behind this study and a source of knowledge to identify outcomes that have been actively promoted in study design. A reduction in perioperative complications, especially haemorrhage, was identified by patients as a main research priority as well as physical and psychosocial wellbeing and quality of life after surgery. The PROM questionnaires were assessed during patient and family interviews to ascertain their accessibility, value and feasibility. The PIS has been reviewed with pre-operative patients and family to obtain feedback on its clarity, readability and content. A transplant recipient patient expert is a co-applicant to this grant and will be an active member of the TMG. Our patient expert will assist us in providing effective communication with patients and public with a patient-centred viewpoint.

Author contribution

For peer review only

Writing and approving manuscript - JF, MS, DM, BD, BS, GA, NW.

Reviewing and approving manuscript - JF, MS, DM, BD, BS, GA, NW.

Funding

This project is funded by the National Institute for Health Research (NIHR) under its Research for Patient Benefit (RfPB) Programme (Grant Reference Number PB-PG-0817-20023). The views expressed are those of the author(s) and not necessarily those of the NIHR or the Department of Health and Social Care.

Competing interests statement

All authors declare that they have no financial or other competing interests.

For peer review only

REFERENCES

1. NHSBT. Annual Report on Liver Transplantation (2019-2020). online] 2020. Accessed 20/04/2021.
2. Rana A, Petrowsky H, Hong JC, et al. Blood transfusion requirement during liver transplantation is an important risk factor for mortality. J Am Coll Surg 2013;216:902-907.
3. de Boer MT, Christensen MC, Asmussen M, et al. The impact of intraoperative transfusion of platelets and red blood cells on survival after liver transplantation. Anesth Analg 2008;106:32-44, table of contents.
4. Reichert B, Kaltenborn A, Becker T, Schiffer M, Klempnauer J, Schrem H. Massive blood transfusion after the first cut in liver transplantation predicts renal outcome and survival. Langenbeck's archives of surgery 2014;399:429-440.
5. D'Amico G, Pagliaro L, Bosch J. Pharmacological Treatment of Portal Hypertension: An Evidence-Based Approach. Semin Liver Dis 1999;19:475-505.
6. Chen X, Ding X, Shen B, et al. Incidence and outcomes of acute kidney injury in patients with hepatocellular carcinoma after liver transplantation. J Cancer Res Clin Oncol 2017.
7. Rahman S, Davidson B, Mallett S. Early acute kidney injury after liver transplantation: Predisposing factors and clinical implications. World J Hepatol 2017.
8. Ayhan A, Ersoy Z, Ulas A, Zeyneloglu P, Pirat A, Haberal M. Incidence and Patient Outcomes in Renal Replacement Therapy After Orthotopic Liver Transplant. Exp Clin Transplant 2017;15:258-260.
9. Zhu M, Li Y, Xia Q, et al. Strong impact of acute kidney injury on survival after liver transplantation. Transplantation proceedings 2010;42:3634-3638.
10. Lima EQ, Zanetta DM, Castro I, et al. Risk factors for development of acute renal failure after liver transplantation. Ren Fail 2003;25:553-560.
11. Yalavarthy R, Edelstein CL, Teitelbaum I. Acute renal failure and chronic kidney disease following liver transplantation. Hemodialysis international International Symposium on Home Hemodialysis 2007;11 Suppl 3:S7-12.
12. Mountokalakis T, Kallivretakis N, Mayopoulou-Symvoulidou D, Karvountzis G, Tolis G. Enhancement of renal function by a long-acting somatostatin analogue in patients with decompensated cirrhosis. Nephrol Dial Transplant 1988;3:604-607.
13. Kaffy F, Borderie C, Chagneau C, et al. Octreotide in the treatment of the hepatorenal syndrome in cirrhotic patients. Journal of hepatology 1999;30:174.
14. Sun H, Zou S, Candiotti KA, et al. Octreotide Attenuates Acute Kidney Injury after Hepatic Ischemia and Reperfusion by Enhancing Autophagy. Sci Rep 2017;7:42701.
15. Sahmeddini MA, Amini A, Naderi N. The effect of octreotide on urine output during orthotopic liver transplantation and early postoperative renal function; a randomized, double-blind, placebo-controlled trial. Hepat Mon 2013;13:e12787.
16. Cacciarelli TV, Keeffe EB, Moore DH, et al. Effect of intraoperative blood transfusion on patient outcome in hepatic transplantation. Arch Surg 1999;134:25-29.
17. Boin IF, Leonardi MI, Luzo AC, Cardoso AR, Caruy CA, Leonardi LS. Intraoperative massive transfusion decreases survival after liver transplantation. Transplantation proceedings 2008;40:789-791.
18. Xia VW, Du B, Braunfeld M, et al. Preoperative characteristics and intraoperative transfusion and vasopressor requirements in patients with low vs. high MELD scores. Liver transplantation : official publication of the American Association for the Study of Liver Diseases and the International Liver Transplantation Society 2006;12:614-620.
19. Garcia-Pagan JC, Escorsell A, Moitinho E, Bosch J. Influence of pharmacological agents on portal hemodynamics: basis for its use in the treatment of portal hypertension. Semin Liver Dis 1999;19:427-438.

For peer review only

20. Ottesen LH, Aagaard NK, Kiszka-Kanowitz M, et al. Effects of a long-acting formulation of octreotide on renal function and renal sodium handling in cirrhotic patients with portal hypertension: a randomized, double-blind, controlled trial. Hepatology 2001;34:471-477.
21. Velez JC, Nietert PJ. Therapeutic response to vasoconstrictors in hepatorenal syndrome parallels increase in mean arterial pressure: a pooled analysis of clinical trials. American journal of kidney diseases : the official journal of the National Kidney Foundation 2011;58:928-938.
22. Mizota T, Hamada M, Matsukawa S, Seo H, Tanaka T, Segawa H. Relationship Between Intraoperative Hypotension and Acute Kidney Injury After Living Donor Liver Transplantation: A Retrospective Analysis. J Cardiothorac Vasc Anesth 2017;31:582-589.
23. Hilmi IA, Damian D, Al-Khafaji A, et al. Acute kidney injury after orthotopic liver transplantation using living donor versus deceased donor grafts: A propensity score-matched analysis. Liver transplantation : official publication of the American Association for the Study of Liver Diseases and the International Liver Transplantation Society 2015;21:1179-1185.
24. Byram SW, Gupta RA, Ander M, Edelstein S, Andreatta B. Effects of Continuous Octreotide Infusion on Intraoperative Transfusion Requirements During Orthotopic Liver Transplantation. Transplantation proceedings 2015;47:2712-2714.
25. Troisi R, Croo A, Codarin E, et al. Somatostatin (Eumedica®) Infusion Allows Modulating Graft Flows and Gradients in Liver Transplantation. Results of a Randomized, Double Blind, Placebo Controlled Trial [abstract]. In: American Transplant Congress; 2015: Am J Transplant, 2015.
26. Chan AW, Tetzlaff JM, Altman DG, et al. SPIRIT 2013 statement: defining standard protocol items for clinical trials. Annals of internal medicine 2013;158:200-207.
27. Moher D, Hopewell S, Schulz KF, et al. CONSORT 2010 explanation and elaboration: updated guidelines for reporting parallel group randomised trials. BMJ 2010;340:c869.
28. Gralnek IM, Hays RD, Kilbourne A, et al. Development and evaluation of the Liver Disease Quality of Life instrument in persons with advanced, chronic liver disease--the LDQOL 1.0. The American journal of gastroenterology 2000;95:3552-3565.
29. EuroQol--a new facility for the measurement of health-related quality of life. Health Policy 1990;16:199-208.
30. Julious SA. Sample size of 12 per group rule of thumb for a pilot study. Pharmaceutical Statistics 2005;4:287-291.
31. Sim J, Lewis M. The size of a pilot study for a clinical trial should be calculated in relation to considerations of precision and efficiency. Journal of clinical epidemiology 2012;65:301-308.
32. Martin D. Optimising the cardiovascular system following liver transplantation surgery. Study code ISRCTN10329248 [online]. Available at: http://www.isrctn.com/ISRCTN10329248.
33. Kamath PS, Kim WR. The model for end-stage liver disease (MELD). Hepatology 2007;45:797-805.
34. Barber K, Madden S, Allen J, Collett D, Neuberger J, Gimson A. Elective liver transplant list mortality: development of a United Kingdom end-stage liver disease score. Transplantation 2011;92:469-476.
35. Collett D, Friend PJ, Watson CJ. Factors Associated With Short- and Long-term Liver Graft Survival in the United Kingdom: Development of a UK Donor Liver Index. Transplantation 2017;101:786-792.

For peer review only

Table 1: World Health Organization Trial Registration Data Set

**Data category**	**Information**
Primary registry and trial identifying number	ClinicalTrials.gov<br>NCT04941911
Date of registration in primary registry	June 28, 2021
Source(s) of monetary or material support	National Institute of Health Research
Primary sponsor	University College London, United Kingdom
Contact for public queries	MS [michaelspiro@nhs.net]
Contact for scientific queries	MS [michaelspiro@nhs.net]
Public title	A Feasibility Study of Octreotide Infusion During Liver Transplant
Scientific title	A Double-blind Randomised Placebo-controlled Feasibility Study to Assess the Impact of Octreotide Infusion During Liver Transplantation on Post-operative Renal Failure
Countries of recruitment	United Kingdom
Health condition(s) or problem(s) studied	Liver transplantation
Intervention(s)	Active comparator: Intravenous Octreotide acetate infusion, 100mcg bolus with a subsequent infusion of 100mcg per hour during surgery
	Placebo comparator: Sodium chloride 0.9% w/v
Key inclusion and exclusion criteria	Ages eligible for study: ≥18 years<br>Sexes eligible for study: both<br>Accepts healthy volunteers: no
	Inclusion criteria: adults aged 18 years and over undergoing primary liver transplantation of a whole or partial liver graft from a cardiac or brain dead donor
	Exclusion criteria: previous solid organ transplant, acute liver

For peer review only

Data category	Information
	failure, fulminant hepatic failure, patients receiving a living donor liver graft, patients currently admitted to ICU prior to transplantation, requirement of haemodialysis or CVVHF pre-operatively, known allergy or adverse reaction to octreotide, pre-operative decision to use intra-operative CVVHF, a positive pregnancy test.
Study type	Interventional
	Allocation: patients will be randomised in a 2:1 ratio to either octreotide or placebo groups. Stratified randomisation of patients by source of liver graft (brain death or cardiac death).<br><br>Masking: quadruple (Participant, Care Provider, Investigator, Outcomes Assessor)
	Primary purpose: feasibility
	Phase II
Date of first enrolment	July 2021
Target sample size	30
Recruitment status	Pending recruitment
Primary outcome(s)	Trial feasibility
Key secondary outcomes	Incidence of acute kidney injury<br><br>Post-operative incidence of a new requirement for renal replacement therapy<br><br>Incidence of new chronic kidney disease or deterioration of chronic kidney disease<br><br>Incidence of early allograft dysfunction<br><br>Patient mortality<br><br>Intra-operative red blood cell salvage<br><br>Volume of packed red blood cell transfusion administered

For peer review only

Data category	Information
	Incidence of adverse events secondary to study drug infusion<br><br>PROMs data

For peer review only

Appendix 1: Study Flowchart

For peer review only

Appendix 2: TMG and TSC terms of reference and functions

Responsibilities of the combined Trial Steering Committee and Data Monitoring Committee

To act as the oversight body for the trial on behalf of the Sponsor and Funder.

The role of the TSC is to provide oversight for the trial and provide advice through its independent Chair to the Chief Investigator, the TMG, trial Sponsor, Funder, and host institution on all aspects of the trial. The rights, safety and well-being of the trial participants are the most important consideration and should prevail over the interests of science and society.

Functions of the TSC include:

- Provide expert oversight of the trial
- Maintain confidentiality of all trial information not already in the public domain
- Review regular progress reports from the trial team
- Monitor recruitment rates and review strategies proposed to deal with any recruitment problems
- Make decisions regarding continuation or termination of the trial
- Assess the impact and relevance of any accumulating external evidence
- Monitor quality of the data and review strategies proposed to improve data collection
- Monitor follow-up rates and review strategies proposed to deal with problems
- Monitor sites that are deviating from the protocol
- Review any proposed amendments to the protocol
- Review proposals by the TMG concerning any change to the design of the trial, including additional sub-studies
- Oversee the timely reporting to trial results
- Review the statistical analysis plan
- Review the publication policy
- Review the main trial manuscript
- Review any abstracts and presentations of results during the trial

Approve external or early internal requests for release of data, subsets of data or samples including clinical data.

For peer review only

Responsibilities of the Trial Management Group

- Input into and comment on the protocol and data collection methods
- Promote the trial
- Develop strategies to encourage recruitment and address any issues with recruitment at participating sites
- Be involved in the day-to-day running of the trial by supporting the CI and trial coordinators
- Provide clinical or other expert guidance to the trial coordinators and participating sites on trial- based matters such as clinical and practical queries and interpretation of information recorded on eCRFs or other data collection tools
- Maintain confidentiality of any trial information that is not in the public domain
- Respond to trial correspondence and any questions in a timely fashion
- Review data collection at sites
- Input into the monitoring and classification of SAEs where clinically appropriate
- Input into the meetings of the TSC, if appropriate
- Provide responses to any issues or concerns raised by TSC
- Be aware of accumulating external evidence and assess its impact and relevance
- Input into the development of the Statistical Analysis Plan where appropriate
- Input into the interpretation and trial report writing
- Report to the TSC regarding trial progress

For peer review only

Appendix 3 Informed Consent Form

Appendix 4 Patient Information Sheet

For peer review only

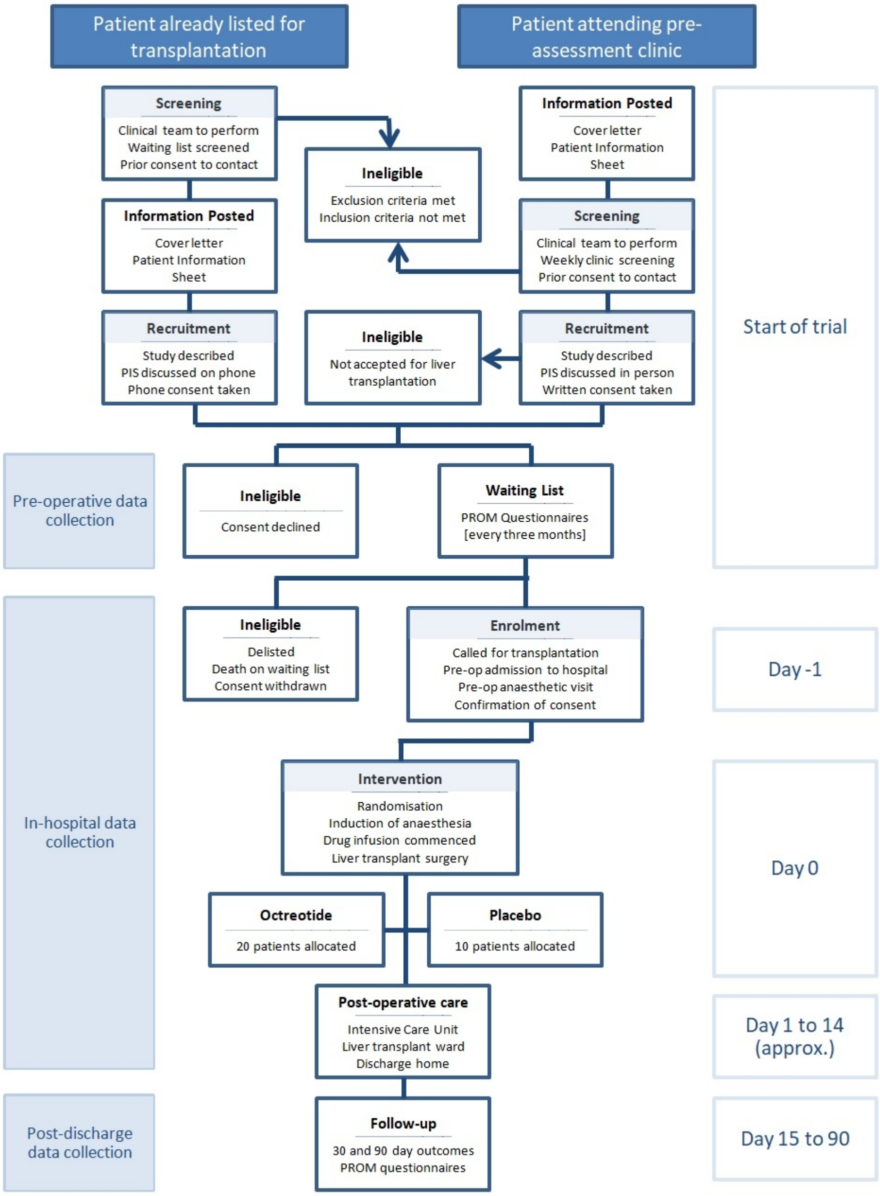

Study Flowchart

248x335mm (96 x 96 DPI)

**Full title of trial**	A double-blind randomised placebo-controlled feasibility study to assess the impact of octreotide infusion during liver transplantation on post-operative renal failure.
**Short title**	A feasibility study of octreotide infusion during liver transplant.

Patient Information Sheet

Study title: A feasibility study of octreotide infusion during liver transplant.

Protocol Reference Number: Sponsor Ref number: 17/0508

We would like to invite you to take part in a research study. Before you decide we would like you to understand why the research is being done and what it would involve for you. One of our team will go through the information sheet with you when you attend hospital for your assessment and answer any questions you have. We would suggest this should take about ten minutes. Talk to others about the study if you wish.

Part 1 tells you the purpose of this study and what will happen to you if you take part. Part 2 gives you more detailed information about the conduct of the study.

Ask us if there is anything that is not clear. Take time to decide whether or not you wish to take part.

<u>Part 1</u>

What is the purpose of the study?

Liver transplant surgery can lead to effects on the kidneys requiring medical support including kidney dialysis. The surgery also has a risk of bleeding and the need for blood transfusion. These complications can slow down recovery after the operation and decrease the chances of the new liver working.

We are investigating a drug called octreotide that is very similar to a natural hormone present in the human body called somatostatin. Octreotide is already used regularly to treat kidney failure in liver disease. We already have evidence that octreotide improves how well the kidney creates urine during and after liver transplant surgery. There is also some evidence that octreotide improves patients' blood pressure in liver disease and during liver transplant surgery and this helps improve kidney function. One published study showed that octreotide might also reduce the amount of blood transfusion required during transplant surgery.

For peer review only - http://bmjopen.bmj.com/site/about/guidelines.xhtml

We are running a research study to find out whether octreotide can improve kidney function during and after transplant surgery and leads to fewer patients needing to have kidney support. We will also look at whether octreotide helps stabilise blood pressure during surgery, reduce bleeding and the need for blood transfusion.

Octreotide is used regularly in some hospitals during liver transplant surgery but it is not clear whether it is beneficial. The quality of research that has been done on octreotide in liver transplant surgery is of poor quality. This research study aims to show if octreotide does have a benefit or not and whether this leads to long-term improvements in patients' health including their survival, the function of the new liver and quality of life.

Why have I been invited?

You have been invited to take part in this clinical trial because you have been referred to either the Royal Free or Queen Elizabeth Hospital for a liver transplant and meet all of the research study criteria for patients to be included. We foresee around thirty patients being involved in this research project over the next two years.

The research team running this clinical trial includes specialists from the liver transplant team, anaesthesia and intensive care as well as liver and kidney specialists.

Is there anything that would stop me from taking part?

If you are undergoing surgery to transplant any organs other than a liver transplant at the same time or if you have previously received any organ transplant, then you would not be able to take part in this study. You would also not be able to take part if you are receiving a 'living donor' liver organ, are suffering from severe liver failure called fulminant hepatic failure or if you have a known allergy to octreotide.

Do I have to take part?

It is up to you to decide to join the study. We will describe the study and go through this information sheet. If you agree to take part, we will then ask you to sign a consent form. You are free to withdraw at any time without giving a reason. Whether or not you are involved in this study, or if you decide to withdraw from the study at any time, will not affect the care you receive. Your involvement in this study will not have any effect on the chances of you receiving a liver transplant.

What will happen to me if I take part?

If you decide to take part then we will ask you to sign an informed written consent form prior to the study procedure.

All of the study takes place in the liver transplant operating theatre while you are under general anaesthetic at the Royal Free or Queen Elizabeth Hospital.

For peer review only

For peer review only - http://bmjopen.bmj.com/site/about/guidelines.xhtml

Your experience of surgery and your care both before and after the transplant procedure will be identical to patients who are not taking part in the study.

In order to find out whether or not octreotide provides a benefit to patients we need to compare patient outcomes between those who have and have not had octreotide. To make the study as effective as possible we will compare the octreotide drug to a placebo. A placebo is a dummy treatment that looks like the real thing but is not. It contains no active ingredient. Patients who agree to be part of this study will receive either an infusion of octreotide or an infusion of an inactive drug (a saline solution).

To make the study as high a quality of research as is possible it will be performed in a 'double blind' fashion. In a 'blind trial' you will not know which treatment group you are in. As this trial is a 'double blind trial', neither you nor your doctor will know in which treatment group you are (although, if your doctor needs to find out he/she can do so).

Sometimes we don't know which way of treating patients is best. To find out, we need to compare different treatments. We put people into groups and give each group a different treatment. The results are compared to see if one is better. To try to make sure the groups are the same to start with, each patient is put into a group by chance (randomly). You have a two-in-three chance of your receiving the active drug (octreotide) in this study, and a one-in-three chance of the inactive drug.

This study is known as a 'feasibility study'. The idea of this type of research study is to find out whether a much larger trial would be possible and how it should be designed. The design of the study is identical to a larger trial but has a smaller number of patients in it.

Neither you nor the doctors looking after you will be aware of if you receive the active drug or not. We will monitor you very closely and record your data throughout your transplant and whilst on intensive care and the ward. The infusion will be started after you are asleep for your liver transplant and be stopped before you are transferred to the intensive care unit.

You will have received this Patient Information Sheet either by post or during your visit to The Royal Free London or Queen Elizabeth Hospital Birmingham. A member of the research team will contact you by phone if you are at home, or visit you whilst you are in the hospital to fully explain this study and offer you the opportunity to be involved. If you wish to take part we will complete a consent form with you when you attend the hospital on the day of your liver transplant.

Your involvement in the research project will be from the time you consent to being involved until ninety days after your liver transplant surgery as we will collect information during your recovery from the transplant procedure.

For peer review only - http://bmjopen.bmj.com/site/about/guidelines.xhtml

When you receive this Patient information leaflet, you will discuss the information with family, friends and/or GP. If interested then contact study team, who will discuss the study with you and you can register an expression of interest and if eligible you will be consented to d be involved in study. If you do not proceed to transplant over the study period you cannot be included in the trial.

The study will involve a small number of questionnaires but will not require you to attend any additional clinics or hospital appointments. These include:

- The completion of an written informed consent form,
- The completion of two quality of life questionnaires (taking roughly thirty minutes) during the pre-operative assessment,
- The completion of two quality of life questionnaires around three months after surgery. These will be done at your routine follow-up appointment and take roughly thirty minutes.

Patients who agree to take part in this study will receive all normal care and treatments for their liver transplant, no treatments will be withheld as part of this study. There is no video or audiotaping or photography involved in this study.

Expenses and payments

There are no expenses or payments involved in this study.

What are the possible disadvantages and risks of taking part?

Octreotide is an artificial version of a hormone that the body produces constantly to help regulate the gut and digestion. Octreotide is used routinely in the NHS for treatment of kidney failure and bleeding in liver disease. Some hospitals use octreotide routinely during liver transplant surgery and feel it provides benefits. Octreotide is a used quite commonly in patients who have liver disease. It is generally regarded as a very safe medication.

However, there are risks with all medical treatments. Effects that have been found from the use of octreotide are:

- Changes in blood sugar levels
- A slowing down in the heart rate
- Changes in heart rhythm

During and after liver transplant surgery the heart rate and rhythm is monitored constantly and if there are any concerns this can be treated. Your blood sugar levels are checked approximately hourly throughout surgery and at a similar frequency

For peer review only - http://bmjopen.bmj.com/site/about/guidelines.xhtml

after surgery on the intensive care unit. If there are concerns regarding your blood sugar levels these are treated routinely.

If at any point during your surgery there is a concern that the medication is causing any problems that cannot be easily treated then the trial medication will be stopped.

There are no risks associated with the placebo infusion.

Before participating you should consider if this will affect any insurance you have and seek advice if necessary.

What are the effects of any treatment received when taking part?

Octreotide has been used in the NHS for a significant period of time and the effects of the medication are well understood.

Octreotide can cause low blood sugar levels in patients with diabetes and increase the effect of drugs taken to control blood sugar levels. Octreotide can also increase blood sugar levels in patients with and without diabetes. All patients in this study will have regular monitoring of blood sugar concentrations throughout their surgery and afterwards on the intensive care unit. All medicines affecting blood sugar levels are stopped routinely before surgery and blood sugar levels are controlled as part of routine care after surgery.

Octreotide may increase the effect of some medications that slow the heart or adjust the heart rhythm. During surgery and while on the intensive care unit the heart rate and rhythm of all patients is monitored continuously as part of routine care and kept in a safe range.

Octreotide can slow down how the body breaks down the medication codeine and this may reduce the pain relief provided by this medication. Codeine is not used as part of routine pain relief for patients who have undergone liver transplant surgery.

Octreotide may decrease the levels of a drug used to suppress the immune system called cyclosporine. Cyclosporin used to be used to reduce the change that the immune system would reject the new liver that had been transplanted. Cyclosporin is no longer used to suppress the immune system in liver transplantation.

There are no effects associated with the placebo infusion.

Anaesthetists, intensive care doctors, and other medical practitioners involved in the care of patients involved in this study will be aware of these possible effects of octreotide. Members of the research and pharmacy team who care for patients on this research study will monitor patients' medications to ensure the chance of effects is made as small as possible.

For peer review only - http://bmjopen.bmj.com/site/about/guidelines.xhtml

We would expect the body to have removed all the octreotide medication within 48 hours after surgery. As such, there will be no concerns regarding octreotide after you have left hospital following your liver transplant surgery.

There are no recognized risks from octreotide to pregnant women. A pregnancy test is required for all women who are at are at risk of pregnancy before they can undergo liver transplant surgery. A positive pregnancy test would prevent you from taking part in this study. If you take part in this study there are no restrictions on you becoming pregnant following your liver transplant surgery, other than those given to you by your clinical care team.

What are the possible benefits of taking part?

We cannot promise the study will help you but the information we get from this study will help improve the treatment of people who are receiving future liver transplants.

Octreotide use during liver transplant surgery is a standard part of care in some of the world's top transplant institutes. The evidence we have so far suggests that octreotide infusions may reduce the chances of kidney failure after surgery, improve blood pressure, reduce bleeding and the need for blood transfusion. As such, if you receive the octreotide infusion your care may benefit, although we cannot be certain.

What happens when the research study stops?

Only a single treatment of octreotide or placebo will be given in this study. There is no evidence to suggest that further doses of octreotide after surgery would be of any benefit.

Your medical care after liver transplant surgery will be identical to all other patients who are not part of this research study. As such, there will be no changes after the research study stops.

It will not be possible to tell patients which medication they were given during the research study.

Once the study has been completed the data will be analysed and this may suggest that we should use Octreotide routinely. If so this could be introduced for future transplant patients. It is more likely that the data will indicate that a larger study is required to demonstrate whether Octreotide is beneficial in this situation.

What if there is a problem?

If you seem to have an adverse reaction to this medication, we will immediately stop the infusion of the trial medication and treat the complication if needed. We will document what has happened and inform you of what has happened.

For peer review only - http://bmjopen.bmj.com/site/about/guidelines.xhtml

Any complaint about the way you have been dealt with during the clinical trial or any possible harm you might suffer will be addressed. The detailed information concerning this is given in Part 2 of this information sheet. If you have any concerns or complaints you should contact your study doctor in the first instance.

Will my taking part in the study be kept confidential?

Yes. We will follow ethical and legal practice and all information about you will be handled in confidence. The details are included in Part 2.

For peer review only

For peer review only - http://bmjopen.bmj.com/site/about/guidelines.xhtml

Contact Details

Your Doctor

Queen Elizabeth Hospital, Birmingham

Consultant Anaesthetist

Royal Free Hospital, London

Consultant Anaesthetist and Intensivist

Your Research Nurse

Insert local contact details

Name

Tel. Number:

This completes Part 1 of the Information Sheet.

If the information in Part 1 has interested you and you are considering participation, please read the additional information in Part 2 before making any decision.

For peer review only - http://bmjopen.bmj.com/site/about/guidelines.xhtml

<u>Part 2</u>

What if relevant new information becomes available?

Sometimes we get new information about the treatment being studied. If this happens, we will tell you about it and discuss whether you want to or should continue in the study. If you decide not to carry on there will be no changes to your normal care before and after your transplant surgery. If you decide to continue in the study we will ask you to sign an updated consent form.

If the study is stopped for any other reason, we will tell you why and ensure your continuing care is unaffected.

What will happen if I don't want to carry on with the study?

You can withdraw from the study at any time without giving a reason, either before or after your liver transplant surgery. It will not affect your future care in any way.

If you choose to withdraw from the study before your liver transplant surgery then you will not be given the trial medication and any data that can be identified as yours can be destroyed if you wish.

If you choose to withdraw from the study after your liver transplant surgery then we would continue to collect and inspect data regarding drug safety and effects. We would also request that your data is used for the study analysis. However, we would not collect any other data and data not relating to this, for instance to do with your quality of life, would be destroyed if you wished. This is because we would have duty of care to you to ensure that you were safe having received the study medication.

What if there is a problem?

If you have a concern about any aspect of this study you should speak directly to the study organiser or a member of the research team. The anaesthetic and intensive care team involved in your care will be able to contact them for you directly. If you wish to complain about any aspect of the way in which you have been approached or treated during the course of this study, you can contact the study doctors through their contact details, below.

You may also wish to contact the Royal Free or Queen Elizabeth Hospital Patient Advice and Liaison Service on 020 7472 6446 (Royal Free) or 0121 424 0808 (Queen Elizabeth Hospital). They offer offers support, information and assistance to patients, relatives and visitors about medical services and hospital care.

Every care will be taken in the course of this clinical trial. However, in the unlikely event that you are injured by taking part, compensation may be available.

For peer review only - http://bmjopen.bmj.com/site/about/guidelines.xhtml

If you suspect that the injury is the result of the Sponsor's (University College London) or the hospital's negligence, then you may be able to claim compensation. After discussing with your study doctor, please make the claim in writing to the Dr. Michael Spiro who is the Chief Investigator for the clinical trial and is based at The Royal Free Hospital, Department of Anaesthesia. The Chief Investigator will then pass the claim to the Sponsor's Insurers, via the Sponsor's office. You may have to bear the costs of the legal action initially, and you should consult a lawyer about this.

Participants may also be able to claim compensation for injury caused by participation in this clinical trial without the need to prove negligence on the part of University College London or another party. You should discuss this possibility with your study doctor in the same way as above.

Regardless of this, if you wish to complain, or have any concerns about any aspect of the way you have been approached or treated by members of staff or about any effects (adverse events) you may have experienced due to your participation in the clinical trial, the normal National Health Service complaints mechanisms are available to you. Please ask your study doctor if you would like more information on this. Details can also be obtained from the Department of Health website: http://www.dh.gov.uk.

Will my taking part in this study be kept confidential?

If you consent to take part in this study, the records obtained while you are in this study as well as related health records will remain strictly confidential at all times. The information will be held securely on paper and electronically at your treating hospital and the main hospital site managing this research (The Royal Free Hospital, London and University Hospitals Birmingham NHS Foundation Trust) under the provisions of the 1998 Data Protection Act. Your name will not be passed to anyone else out the research team or the Sponsor (UCL), who is not involved in the trial. You will be allocated a trial number, which will be used as a code to identify you on all trial forms. Any information about you which leaves the hospital/surgery will have your name and address removed so that you cannot be recognised.

Your records will be available to people authorised to work on the trial but may also need to be made available to people authorised by the Sponsor, which is the organisation responsible for ensuring that the study is carried out correctly. By signing the consent form you agree to this access for the current study and any further research that may be conducted in relation to it, even if you withdraw from the current study. Data collected during this phase of the study will be included in a larger follow-on study performed in an identical fashion.

The information collected about you may also be shown to authorised people from the UK Regulatory Authority (the Medicines and Healthcare Products Regulatory Authority); this is to ensure that the study is carried out to the highest possible scientific standards. All will have a duty of confidentiality to you as a research participant.

For peer review only - http://bmjopen.bmj.com/site/about/guidelines.xhtml

If you withdraw consent from further study treatment, unless you object, your data and samples will remain on file and will be included in the final study analysis.

In line with the regulations, at the end of the study your data will be securely archived for a minimum of 20 years. Arrangements for confidential destruction will then be made.

Anonymised Data collected during the study may be transferred for the purpose of analysis to associated researchers within/out the European Economic Area. Some countries outside of Europe may not have laws which protect your privacy to the same extent as the Data Protection Act in the UK or European Law. The Sponsor of the trial will take all reasonable steps to protect your privacy.

Will my GP be informed of my involvement?

With your permission, your GP, and other doctors who may be treating you, will be notified that you are taking part in this study.

What will happen to any samples I give?

No samples will be taken.

Will any genetic tests be done?

No genetic tests will be performed.

What will happen to the results of the research study?

The results of the study will be available after it finishes and will usually be published in a medical journal or be presented at a scientific conference. The data will be anonymous and none of the patients involved in the trial will be identified in any report or publication.

Should you wish to see the results, or the publication, please ask your study doctor. We will send all interested patients a copy of the study results and will arrange a presentation of the research findings to the participants and the wider liver transplant community.

Who is organising and funding the research?

The research is being funded by the National Institute for Health Research as part of their Research for Patient Benefit programme. The doctors involved in setting up and running this research project are not being paid to do so. The doctors and wider research team do not have any conflict of interests in this study and no companies or members of the pharmaceutical industry are involved in any way.

Who has reviewed the study?

For peer review only - http://bmjopen.bmj.com/site/about/guidelines.xhtml

All research in the NHS is looked at by independent group of people, called a Research Ethics Committee, to protect your interests. This study has been reviewed and given favourable opinion by East Midlands - Leicester South Research Ethics Committee.

Further information and contact details

You are encouraged to ask any questions you wish, before, during or after your treatment. If you have any questions about the study, please speak to your study nurse or doctor, who will be able to provide you with up to date information about the drug(s)/procedure(s) involved. If you wish to read the research on which this study is based, please ask your study nurse or doctor. If you require any further information or have any concerns while taking part in the study, please contact one of the following people:

Your Doctor

Queen Elizabeth Hospital, Birmingham

Consultant Anaesthetist

Royal Free Hospital, London

Consultant Anaesthetist and Intensivist

Your Research Nurse

Name

Tel. Number:

Alternatively, if you or your relatives have any questions about this study you may wish to contact one of the following organisations that are independent of the hospital at which you are being treated:

If you decide you would like to take part then please read and sign the consent form. You will be given a copy of this information sheet and the consent form to keep. A copy of the consent form will be filed in your patient notes, one will be filed with the study records and one may be sent to the Research Sponsor.

You can have more time to think this over if you are at all unsure.

For peer review only - http://bmjopen.bmj.com/site/about/guidelines.xhtml

Thank you for taking the time to read this information sheet and to consider this study.

For peer review only - http://bmjopen.bmj.com/site/about/guidelines.xhtml

Study Number: IRAS 278918
Sponsor Reference number 17/0508
Centre Number:
Patient Identification Number/Subject Number for this trial:

INFORMED CONSENT FORM (ICF)

In-Person Consent

Project Title Assessing the Impact of Octreotide Infusion during Liver Transplantation

Confirmation of consent to be performed on admission for transplantation<br>Points one to seven to be confirmed<br>Patient confirmation signature overleaf

Please initial box

1. I confirm that I have read and understand the information sheet dated 15.01.2021 (version 1.0) for the above study. I have had the opportunity to consider the information, ask questions and have had these answered satisfactorily. ☐

2. I understand that my participation is voluntary and that I am free to withdraw at any time without giving any reason, without my medical care or legal rights being affected. ☐

3. I understand that relevant sections of my medical notes and data collected during the study, may be looked at by individuals from the sponsor of the trial (University College London) and responsible persons authorised by the sponsor, from regulatory authorities or from the NHS Trust, where it is relevant to my taking part in this research. I give permission for these individuals to have access to my records. ☐

4. I agree to my GP being informed of my participation in the study. ☐

5. I consent to the collection, processing, reporting, storage and transfer within and outside Europe of my anonymised data for healthcare and/or medical research purposes in relation to this research. I understand that I will not be directly identifiable except to the study doctor and his/her study team. ☐

6. I agree to take part in the above study. ☐

7. After the entire study has completed I would like to receive a letter indicating which medication (octreotide or placebo) I received. [Optional]. ☐

For peer review only - http://bmjopen.bmj.com/site/about/guidelines.xhtml

Name of patient	Date	Signature
Name of person taking consent	Date	Signature
Name of Chief Investigator *(if different to the person taking consent)*	Date	Signature

When completed: 1 for participant; 1 (original) for researcher site file; 1 to be kept in medical notes. Please file a copy of the GP letter in the medical records and write a note in the medical record stating when the patient consented and date when the GP letter was sent.

Confirmation of consent on admission for transplantation

Name of patient	Date	Signature
Name of person taking consent	Date	Signature

For peer review only - http://bmjopen.bmj.com/site/about/guidelines.xhtml

Reporting checklist for protocol of a clinical trial

		Reporting Item	Page Number
**Administrative information**			
Title	#1	Descriptive title identifying the study design, population, interventions, and, if applicable, trial acronym	1
Trial registration	#2a	Trial identifier and registry name. If not yet registered, name of intended registry	2
Trial registration: data set	#2b	All items from the World Health Organization Trial Registration Data Set	16-19
Protocol version	#3	Date and version identifier	2
Funding	#4	Sources and types of financial, material, and other support	2
Roles and responsibilities: contributorship	#5a	Names, affiliations, and roles of protocol contributors	1
Roles and responsibilities: sponsor contact information	#5b	Name and contact information for the trial sponsor	3

For peer review only

Roles and responsibilities: sponsor and funder	#5c	Role of study sponsor and funders, if any, in study design; collection, management, analysis, and interpretation of data; writing of the report; and the decision to submit the report for publication, including whether they will have ultimate authority over any of these activities	3
Roles and responsibilities: committees	#5d	Composition, roles, and responsibilities of the coordinating centre, steering committee, endpoint adjudication committee, data management team, and other individuals or groups overseeing the trial, if applicable (see Item 21a for data monitoring committee)	3
**Introduction**			
Background and rationale	#6a	Description of research question and justification for undertaking the trial, including summary of relevant studies (published and unpublished) examining benefits and harms for each intervention	4-5
Background and rationale: choice of comparators	#6b	Explanation for choice of comparators	4-5
Objectives	#7	Specific objectives or hypotheses	5
Trial design	#8	Description of trial design including type of trial (eg, parallel group, crossover, factorial, single group), allocation ratio, and framework (eg, superiority, equivalence, non-inferiority, exploratory)	5

For peer review only

**Methods: Participants, interventions, and outcomes**			
Study setting	#9	Description of study settings (eg, community clinic, academic hospital) and list of countries where data will be collected. Reference to where list of study sites can be obtained	5
Eligibility criteria	#10	Inclusion and exclusion criteria for participants. If applicable, eligibility criteria for study centres and individuals who will perform the interventions (eg, surgeons, psychotherapists)	5-6
Interventions: description	#11a	Interventions for each group with sufficient detail to allow replication, including how and when they will be administered	7
Interventions: modifications	#11b	Criteria for discontinuing or modifying allocated interventions for a given trial participant (eg, drug dose change in response to harms, participant request, or improving / worsening disease)	7
Interventions: adherance	#11c	Strategies to improve adherence to intervention protocols, and any procedures for monitoring adherence (eg, drug tablet return; laboratory tests)	7-8
Interventions:	#11d	Relevant concomitant care and interventions that are	7

For peer review only

concomitant care		permitted or prohibited during the trial	
Outcomes	#12	Primary, secondary, and other outcomes, including the specific measurement variable (eg, systolic blood pressure), analysis metric (eg, change from baseline, final value, time to event), method of aggregation (eg, median, proportion), and time point for each outcome. Explanation of the clinical relevance of chosen efficacy and harm outcomes is strongly recommended	8-9
Participant timeline	#13	Time schedule of enrolment, interventions (including any run-ins and washouts), assessments, and visits for participants. A schematic diagram is highly recommended (see Figure)	20
Sample size	#14	Estimated number of participants needed to achieve study objectives and how it was determined, including clinical and statistical assumptions supporting any sample size calculations	10
Recruitment	#15	Strategies for achieving adequate participant enrolment to reach target sample size	6
**Methods: Assignment of interventions (for controlled trials)**			
Allocation: sequence generation	#16a	Method of generating the allocation sequence (eg, computer-generated random numbers), and list of any factors for stratification. To reduce predictability of a	8

For peer review only

		random sequence, details of any planned restriction (eg, blocking) should be provided in a separate document that is unavailable to those who enrol participants or assign interventions	
Allocation concealment mechanism	#16b	Mechanism of implementing the allocation sequence (eg, central telephone; sequentially numbered, opaque, sealed envelopes), describing any steps to conceal the sequence until interventions are assigned	8
Allocation: implementation	#16c	Who will generate the allocation sequence, who will enrol participants, and who will assign participants to interventions	8
Blinding (masking)	#17a	Who will be blinded after assignment to interventions (eg, trial participants, care providers, outcome assessors, data analysts), and how	8
Blinding (masking): emergency unblinding	#17b	If blinded, circumstances under which unblinding is permissible, and procedure for revealing a participant's allocated intervention during the trial	8
**Methods: Data collection, management, and analysis**			
Data collection plan	#18a	Plans for assessment and collection of outcome, baseline, and other trial data, including any related processes to promote data quality (eg, duplicate measurements, training	10

For peer review only

		of assessors) and a description of study instruments (eg, questionnaires, laboratory tests) along with their reliability and validity, if known. Reference to where data collection forms can be found, if not in the protocol	
Data collection plan: retention	#18b	Plans to promote participant retention and complete follow-up, including list of any outcome data to be collected for participants who discontinue or deviate from intervention protocols	10-11
Data management	#19	Plans for data entry, coding, security, and storage, including any related processes to promote data quality (eg, double data entry; range checks for data values). Reference to where details of data management procedures can be found, if not in the protocol	10, 12
Statistics: outcomes	#20a	Statistical methods for analysing primary and secondary outcomes. Reference to where other details of the statistical analysis plan can be found, if not in the protocol	10-11
Statistics: additional analyses	#20b	Methods for any additional analyses (eg, subgroup and adjusted analyses)	11
Statistics: analysis population and missing data	#20c	Definition of analysis population relating to protocol non-adherence (eg, as randomised analysis), and any statistical methods to handle missing data (eg, multiple imputation)	11
**Methods: Monitoring**			
Data monitoring:	#21a	Composition of data monitoring committee (DMC);	11-12,

For peer review only

formal committee		summary of its role and reporting structure; statement of whether it is independent from the sponsor and competing interests; and reference to where further details about its charter can be found, if not in the protocol. Alternatively, an explanation of why a DMC is not needed	21-22
Data monitoring: interim analysis	#21b	Description of any interim analyses and stopping guidelines, including who will have access to these interim results and make the final decision to terminate the trial	12
Harms	#22	Plans for collecting, assessing, reporting, and managing solicited and spontaneously reported adverse events and other unintended effects of trial interventions or trial conduct	12
Auditing	#23	Frequency and procedures for auditing trial conduct, if any, and whether the process will be independent from investigators and the sponsor	12
**Ethics and dissemination**			
Research ethics approval	#24	Plans for seeking research ethics committee / institutional review board (REC / IRB) approval	12
Protocol amendments	#25	Plans for communicating important protocol modifications (eg, changes to eligibility criteria, outcomes, analyses) to relevant parties (eg, investigators, REC / IRBs, trial participants, trial registries, journals, regulators)	12-13

For peer review only

Consent or assent	#26a	Who will obtain informed consent or assent from potential trial participants or authorised surrogates, and how (see Item 32)	6
Consent or assent: ancillary studies	#26b	Additional consent provisions for collection and use of participant data and biological specimens in ancillary studies, if applicable	6
Confidentiality	#27	How personal information about potential and enrolled participants will be collected, shared, and maintained in order to protect confidentiality before, during, and after the trial	13
Declaration of interests	#28	Financial and other competing interests for principal investigators for the overall trial and each study site	2
Data access	#29	Statement of who will have access to the final trial dataset, and disclosure of contractual agreements that limit such access for investigators	10
Ancillary and post trial care	#30	Provisions, if any, for ancillary and post-trial care, and for compensation to those who suffer harm from trial participation	13
Dissemination policy: trial results	#31a	Plans for investigators and sponsor to communicate trial results to participants, healthcare professionals, the public, and other relevant groups (eg, via publication, reporting in results databases, or other data sharing arrangements), including any publication restrictions	13

For peer review only

Dissemination policy: authorship	#31b	Authorship eligibility guidelines and any intended use of professional writers	13
Dissemination policy: reproducible research	#31c	Plans, if any, for granting public access to the full protocol, participant-level dataset, and statistical code	10, 13

Appendices

Informed consent materials	#32	Model consent form and other related documentation given to participants and authorised surrogates	N/A
Biological specimens	#33	Plans for collection, laboratory evaluation, and storage of biological specimens for genetic or molecular analysis in the current trial and for future use in ancillary studies, if applicable	N/A

Notes:

- 21a: 11-12, 21-22 The SPIRIT Explanation and Elaboration paper is distributed under the terms of the Creative Commons Attribution License CC-BY-NC. This checklist was completed on 30. June 2021 using https://www.goodreports.org/, a tool made by the EQUATOR Network in collaboration with Penelope.ai

For peer review only

BMJ Open

Protocol for a prospective double-blind, randomised, placebo-controlled feasibility trial of octreotide infusion during liver transplantation

Journal:	*BMJ Open*
Manuscript ID	bmjopen-2021-055864.R1
Article Type:	Protocol
Date Submitted by the Author:	13-Oct-2021
Complete List of Authors:	Fabes, Jez; University of Plymouth, Peninsula Medical School; Royal Free London NHS Foundation Trust, Department of Anaesthesia<br>Ambler, Gareth; University College London, Department of Statistical Science<br>Shah, Bina; University College London Division of Surgery & Interventional Science<br>Williams, Norman; University College London, UCL Division of Surgery & Interventional Science<br>Martin, Daniel; University of Plymouth, Peninsula Medical School; University College London, Division of Surgery<br>Davidson, Brian; University College London, Division of Surgery<br>Spiro, Michael; University College London, Division of Surgery
**Primary Subject Heading**:	Anaesthesia
Secondary Subject Heading:	Gastroenterology and hepatology, Qualitative research, Surgery
Keywords:	Adult anaesthesia < ANAESTHETICS, Hepatobiliary disease < GASTROENTEROLOGY, Adult intensive & critical care < INTENSIVE & CRITICAL CARE, Hepatology < INTERNAL MEDICINE, Hepatobiliary surgery < SURGERY, Transplant surgery < SURGERY

SCHOLARONE™
Manuscripts

I, the Submitting Author has the right to grant and does grant on behalf of all authors of the Work (as defined in the below author licence), an exclusive licence and/or a non-exclusive licence for contributions from authors who are: i) UK Crown employees; ii) where BMJ has agreed a CC-BY licence shall apply, and/or iii) in accordance with the terms applicable for US Federal Government officers or employees acting as part of their official duties; on a worldwide, perpetual, irrevocable, royalty-free basis to BMJ Publishing Group Ltd ("BMJ") its licensees and where the relevant Journal is co-owned by BMJ to the co-owners of the Journal, to publish the Work in this journal and any other BMJ products and to exploit all rights, as set out in our licence.

The Submitting Author accepts and understands that any supply made under these terms is made by BMJ to the Submitting Author unless you are acting as an employee on behalf of your employer or a postgraduate student of an affiliated institution which is paying any applicable article publishing charge ("APC") for Open Access articles. Where the Submitting Author wishes to make the Work available on an Open Access basis (and intends to pay the relevant APC), the terms of reuse of such Open Access shall be governed by a Creative Commons licence – details of these licences and which Creative Commons licence will apply to this Work are set out in our licence referred to above.

Other than as permitted in any relevant BMJ Author's Self Archiving Policies, I confirm this Work has not been accepted for publication elsewhere, is not being considered for publication elsewhere and does not duplicate material already published. I confirm all authors consent to publication of this Work and authorise the granting of this licence.

For peer review only

Title

Protocol for a prospective double-blind, randomised, placebo-controlled feasibility trial of octreotide infusion during liver transplantation

J. Fabes[1], G. Ambler[2], B. Shah[2], N. Williams[2], D. Martin[1], B. Davidson[3], M. Spiro[3]

1 University

Corresponding author

Dr. Jez Fabes, jeremy.fabes@nhs.net

For peer review only

Abstract

Introduction

Liver transplantation is a complex operation that can provide significant improvements in quality of life and survival to the recipients. However, serious complications are common and include major haemorrhage, hypotension and renal failure. Blood transfusion and the development of acute kidney injury lead to both short- and long-term poor patient outcomes, including an increased risk of death, graft failure, length of stay and reduced quality of life. Octreotide may reduce the incidence of renal dysfunction, perioperative haemorrhage and enhance intraoperative blood pressure. However, octreotide does have risks, including resistant bradycardia, hyper- and hypoglycaemia and QT prolongation. Hence, a randomised controlled trial of octreotide during liver transplantation is needed to determine the cost-efficacy and safety of its use; this study represents a feasibility study prior to this trial.

Methods and analysis

We describe a multi-centre, double-blind, randomised, placebo-controlled feasibility study of continuous infusion of octreotide during liver transplantation surgery. We will recruit 30 adult patients at two liver transplant centres. A blinded infusion during surgery will be administered in a 2:1 ratio of octreotide:placebo. The primary outcomes will determine the feasibility of this study design. These include the recruitment ratio, correct administration of blinded study intervention, adverse event rates, patient and clinician enrolment refusal and completion of data collection. Secondary outcome measures of efficacy and safety will help shape future trials by assessing potential primary outcome measures and monitoring safety end-points. No formal statistical tests are planned. This manuscript represents study protocol number 1.3, dated 2nd June 2021.

Ethics and dissemination

This study has received Research Ethics Committee approval. The main study outcomes will be submitted to an open-access journal.

Registration

The study is registered with ClinicalTrials.gov (NCT04941911) with recruitment due to start in August 2021 with anticipated completion in July 2022. WHO Trial Registration Data Set in Table 1.

Declaration of interests

The chief and principal investigators declare that they have no financial or other competing interests in this study.

Funding

This project is funded by the National Institute for Health Research (NIHR) under its Research for Patient Benefit (RfPB) Programme (Grant Reference Number PB-PG-0817-20023). The views expressed are those of the author(s) and not necessarily those of the NIHR or the Department of Health and Social Care.

For peer review only

Trial Sponsor

The Joint Research Office, University College London, United Kingdom

Neither the Sponsor nor the Funder have any role in study design, collection, management, analysis, and interpretation of data, writing of the study report or the decision to submit the report for publication.

Clinical trials unit

Surgical and Interventional Group, Division of Surgery & Interventional Science, University College London

Key words

Liver transplantation; octreotide; renal failure; patient reported outcome measures; feasibility.

Strengths and limitations of this study

- This study is prospective, randomised and designed to minimise the risk of bias throughout
- We have prioritised patient input throughout the development and design of this study
- A small sample size has been selected to demonstrate feasibility end points as a precursor to a larger efficacy and safety trial
- The secondary end points will provide clinically relevant data on which to design this cost effectiveness study

For peer review only

INTRODUCTION

Liver transplantation is a potentially life transforming intervention with excellent outcomes (one and five year survival of 94% and 84%, respectively[1]). It is, however, a major operation in patients with multiple co-morbidities and is associated with a 3% perioperative mortality rate and 70% complication rate. Intra-operative haemorrhage is serious and commonly occurs during mobilisation of the diseased liver, exacerbated by coagulopathy and abnormal vasculature due to portal hypertension. Bleeding can cause hypotension, reduced perfusion of the transplanted graft and increased incidence of acute kidney injury (AKI). Blood loss and transfusion are associated with increased morbidity, mortality and cost of transplantation and can have a long term detrimental effect on patient wellbeing and quality of life[2-4].

Octreotide is a synthetic analogue of somatostatin with an excellent safety profile[5] that may improve renal outcomes, reduce perioperative bleeding and increase exogenous vasopressor sensitivity during liver transplantation. Acute kidney injury following liver transplantation occurs in 25-60% of patients with normal renal function prior to surgery, with half of these patients requiring renal replacement therapy (RRT) after surgery[6, 7]. The need for post-transplant RRT increases the length of hospital stay and the risk of early allograft dysfunction, chronic kidney disease (CKD) and mortality[8, 9]. Risk factors for renal dysfunction following transplantation include intraoperative hypotension, lower quality donor grafts, hepatic ischemia-reperfusion injury, blood transfusion and pre-operative renal disease[10, 11]. Octreotide has been shown to enhance renal blood flow, glomerular filtration and urine output in decompensated liver cirrhosis[12], hepatorenal syndrome[13] and may also reduce the incidence of post-transplant renal injury[14, 15].

Major haemorrhage and transfusion during and after liver transplantation reduce patient and graft survival as well as increasing length of stay and the need for RRT[2-4, 16-18]. Haemorrhage risk during liver transplantation is multifactorial, including cirrhotic coagulopathy, portal hypertension and hyperfibrinolysis. Bleeding is driven by the presence of wide-spread dilated collaterals and a hyperdynamic splanchnic circulation. Octreotide causes splanchnic vasoconstriction thereby reducing splanchnic blood flow, collateralisation and portal venous pressures which may reduce surgical blood loss[19]. This correction of the abnormal blood distribution and venous pooling increases systemic blood pressure[20] and, when combined with a vasopressor, correlates with improved renal function[21]. Furthermore, increased systemic pressures and vasopressor sensitivity help preserve renal function and reduce myocardial injury[10, 22, 23].

Previous clinical studies of octreotide in liver transplantation

A retrospective cohort study of octreotide infusion during liver transplantation demonstrated a reduction in transfusion requirement by 2.3 units of packed red blood cells but this difference was not statistically significant[24]. A randomised controlled trial (RCT) of octreotide infusion during liver transplantation found an enhancement of noradrenaline effect on blood pressure with a mean increase of 8mmHg[15].

Risks

Octreotide is not currently licensed for use in liver transplantation. Common risks of octreotide include changes in blood sugar levels, heart rate and rhythm. The side effect profile of octreotide

For peer review only

during liver transplantation is unknown. The studies to date did not compare safety or adverse events between the octreotide and placebo arms[15], have not been fully published[25] or did not assess adverse events[24].

Rationale for this study

The background literature for the use of octreotide in liver transplantation is inconclusive and the current use in this setting is clinician and institution dependent. While there is reasonable evidence to support its use in this setting, octreotide has harmful effects which need to be considered against its efficacy and cost. Furthermore, patients may be unwilling to have non-essential medication and clinicians may not support its use in all transplant settings. In addition to determining the feasibility of a multi-site drug trial in liver transplantation, secondary end points will provide clinically relevant data on which to design a subsequent cost-effectiveness study.

Hypothesis

It is feasible to recruit and retain patients in a randomised study of continuous infusion of octreotide during liver transplantation surgery.

METHODS AND ANALYSIS

Trial design

The purpose of this study is to determine the feasibility of recruiting patients to a double-blind, randomised, placebo-controlled trial of intravenous octreotide during liver transplantation. We will recruit 30 adult patients at two liver transplant centres; The Royal Free London NHS Foundation Trust (Royal Free Hospital, RFH), and University Hospitals Birmingham NHS Foundation Trust (University Hospital Birmingham, UHB). A blinded infusion will be administered during transplantation surgery. Patients will be allocated in a 2:1 ratio to octreotide or placebo. All members of the research, anaesthetic, surgical, intensive care and nursing teams will be blinded to patient allocation; this includes all outcome assessors and data analysts. Patients will be blinded to their allocation and will have the option to find out which intervention they received after the study is complete. Unblinding will be carried out once data cleaning and analysis has been completed.

This protocol conforms to the Standard Protocol Items: Recommendations for Interventional Trials (SPIRIT)[26]. The RCT will be reported in line with the Consolidated Standards of Reporting Trials (CONSORT) statement[27]. This trial is non-commercial and does not intend to generate data for drug licensing. The study is registered with ClinicalTrials.gov (NCT04941911) with recruitment due to start in August 2021 with anticipated completion in July 2022.

Eligibility

Inclusion criteria

1. Adults aged 18 years and over accepted for liver transplant and on the transplant waiting list.
2. Undergoing liver transplantation.
3. Recipient of a whole or partial liver graft from a cardiac or brain dead donor.

For peer review only

Exclusion criteria

1. Lacking capacity to provide consent (according to the Mental Capacity Act, 2005).
2. Recipients of multiple solid organ transplants.
3. Previous receipt of a solid organ transplant.
4. Patients with acute or fulminant hepatic failure.
5. Patients receiving a live-donor liver graft.
6. Patients admitted to an intensive care unit (ICU) at the time of transplantation.
7. Preoperative requirement for RRT, including dialysis.
8. Known allergy or adverse reaction to octreotide.
9. Preoperative decision to use intraoperative RRT.
10. Patients enrolled in another interventional study that affects patient care during transplantation surgery or would be expected to have an effect on the study outcomes will be excluded from this study.

Patients who are enrolled in a study examining the impact of extracorporeal machine perfusion of the donated liver prior to transplantation will be eligible for this study.

Patient identification and recruitment

Participant screening and registration will be undertaken locally at each study site. Overall trial flow is outlined in Appendix 1.

Patients will be identified and recruited in two ways, depending on whether they are undergoing assessment for suitability for liver transplantation or have already been accepted onto the waiting list for transplantation. Screening will be performed by the clinical team, as will primary contact regarding engagement with research, prior to research team contact.

i. Patients undergoing assessment for liver transplantation will be sent the patient information sheet (PIS) for this study. Patients that meet the inclusion criteria will be approached during their in-patient assessment process to explain the study and for preliminary informed consent.

ii. Patients already listed for liver transplantation who meet the inclusion criteria will be sent the PIS. These patients will be contacted by phone, the study and PIS explained, and preliminary informed consent taken.

Due to the waiting time for transplant and the fixed duration of this study many patients who would be willing to enter the study will not be able to participate because a donor organ does not become available. Both groups of patients will therefore have their consent confirmed on the day of admission for surgery at which point, once the transplant surgery has been confirmed, the patient will be considered to be enrolled.

Consent

Informed consent will be obtained by a member of the research team and confirmed when the patient is admitted for surgery. The PIS and consent form (Appendices 2 and 3) will be reviewed and updated, if necessary, throughout the trial (e.g. where new safety information becomes available). Consent will be sought for use of participant data in further studies in relation to this research.

For peer review only

Study interventions

Patients will follow the standard protocol for liver transplantation at each study centre with the only study intervention being the provision of octreotide or placebo infusion. The study infusion will commence after induction of anaesthesia and prior to surgical incision. There will be no cross-over or dose escalation. The study infusate will be administered intravenously through an electronic infusion pump until completion of 50 ml of study medication or transfer to ICU, whichever is sooner. The maximum total dose of octreotide infused will be 1 mg, including an initial slow bolus followed by an infusion. Similarly, the maximum dose of 0.9% saline placebo administrated will be 50 ml. Where the infusion is completed prior to the end of surgery then no further study drug or placebo will be given.

Intervention group

Syringes will contain 50ml of octreotide acetate at 20mcg/ml in 0.9% w/v sodium chloride in water.

The octreotide infusion will commence with a 100 mcg bolus (5 ml) over 30 minutes with a subsequent infusion of 100 mcg (5 ml) per hour during surgery.

Comparator group

Syringes will contain 50ml of 0.9% w/v sodium chloride in water.

The control infusion will commence with a 5 ml bolus over 30 minutes with a subsequent infusion of 5 ml per hour during surgery.

Study drug

Octreotide is licensed in the UK and is being used outside of its marketed authorisation for the purpose of this trial. The Summary of Product Characteristics will be used as a safety reference and the dose will not exceed the maximum dose of 1.5 mg per day. A suitable marketed product will be sourced by the RFH Pharmacy Manufacturing Unit that allows seven days stability after reconstitution. The study drug will be prepared and released by a Qualified Person for use in the study by the holder of a Manufacturing and Import Authorisation (Investigational Medicinal Products) licence.

Overdose

Deviations from the treatment schedule or inadvertent administration of additional octreotide may lead to overdose. These will be reported in the case report form (CRF), the patient will be notified and the information and implications reported to the Sponsor. The patient would continue to be included in the trial. Where a serious adverse event (SAE) occurs in association with an overdose then the nature, dose and cause of the overdose will be included in an SAE report form. Participants who receive an overdose of octreotide will be followed up and treated in the usual manner for the trial.

Relevant symptoms of overdose include arrhythmia, hypotension, cardiac arrest, pancreatitis, hepatic steatosis, diarrhoea and lactic acidosis. Management is supportive.

For peer review only

Randomisation

Consented patients who proceed to transplantation will be randomised. Remote-site computerised random allocation will be performed with a centrally generated code (www.sealedenvelope.com) that will correlate with a coded syringe held in the secure study drug storage facility at each study centre. Patients will be stratified within the randomisation process by source of liver graft (brain death, DBD, or cardiac death donor, DCD) to ensure that patients are balanced for these factors across the two treatment groups. Patients will be randomly allocated to either the octreotide or placebo study groups in a 2:1 ratio. The randomisation list will be held centrally at a dedicated and independent off-site facility that will be contactable 24 hours a day.

Allocation concealment

The syringes of octreotide 20 mcg/ml (intervention) and sodium chloride 0.9% w/v (control) will appear identical and only identifiable by their trial-allocated code. No members of the research or clinical team will be aware of patient allocation or syringe contents. The allocation code will only be broken following completion of patient follow up, data cleaning and analysis; unless an emergency break code is requested. The study statistician will be provided only with information on treatment A or B.

Unblinding

Where required for medical or safety reasons a trial break code can be obtained from a nominated member of the research team who will remain blinded to patient allocation. Information on the indication and outcome of the unblinding will be forwarded to the Trial Steering Committee (TSC) and included in the final trial report. While Suspected Unexpected Serious Adverse Reaction (SUSAR) reports will include the treatment allocation, this information will not be available to the trial team and blinding will be maintained.

Outcomes

Primary outcome

The primary outcomes for this study are based on determining the feasibility of conducting a randomised controlled trial of octreotide versus placebo infusion during liver transplantation. The feasibility will be assessed by;

- Recruitment ratio (percentage of eligible patients undergoing transplantation who provide consent, target: ≥ 30%),
- Percentage of eligible patients who receive the study drug infusion in a blinded manner (target: ≥ 80% of patients),
- Incidence of drug-related serious unexpected adverse events (target: ≤ 20%),
- Completion of follow-up data collection (target: ≥ 90% patients),
- Patient refusal to enrol in study on admission for transplantation (target: ≤ 15%),
- Clinician refusal for inclusion or randomisation following patient recruitment (target: ≤ 15%).

Secondary outcomes

For peer review only

A number of secondary outcome measures will be collected to help shape future trials in terms of assessing potential primary outcome measures and monitoring safety end-points.

- Intra-operative cell salvage volume (as a proxy for blood loss),
- Intra-operative urine output during each surgical phase,
- Mean arterial blood pressure during each surgical phase,
- Requirement for and mean vasopressor and/or inotrope infusion rate during each surgical phase,
- Volume or mass of *de novo* packed red blood cells and other clotting products during surgery and on the ICU (within first 24 hours, 72 hours and one week),
- Incidence of AKI as defined by the Acute Kidney Injury Network stage 1 criteria (a 50% increase in serum creatinine from baseline or less than 0.5 ml/kg/hr urine output for 6-12 hours post-transplant) within 24 hours, 72 hours, one week,
- Incidence of postoperative RRT at 24 hours, 72 hours, one and two weeks postoperatively,
- Incidence of new or worsened CKD at thirty and ninety days,
- Mortality, early allograft dysfunction, graft loss and liver graft function at thirty and ninety days,
- Patient recorded outcome measures (PROM) preoperatively and at ninety days postoperatively;
    - Liver Disease Quality of Life questionnaire[28],
    - EuroQOL-5D-5L questionnaire[29].

Major safety outcomes will include;

- Unexpected or resistant bradycardia,
- Abnormal QTc interval (460ms in men, 470ms in women) or associated ventricular arrhythmia or Torsades de Pointes,
- Unexpected or resistant hypoglycaemia (blood sugar < 4 mmol/L),
- Potential allergic or anaphylactic reaction to any medication,
- Any report of an abnormal response to codeine or morphine,
- Development of venous or arterial thrombosis,
- Cardiac event including acute coronary syndrome, new heart failure, arrhythmia or resuscitated cardiac arrest.

Participant withdrawal

A participant may be withdrawn from the trial by their clinician whenever continued participation is considered to no longer be in the participant's best interests, but the reasons for doing so must be recorded. In these cases routine follow-up and safety data will be collected but not any further study-specific data.

A participant may withdraw from the trial at any point without impact on their ongoing care. No further data would be collected. However, data up to that point will be retained. Permission will be sought from patients wishing to withdraw to allow use of their routine follow-up data for trial purposes, especially safety data.

For peer review only

Sample size

Thirty patients will be recruited from two sites, with 20 in the study drug (octreotide) arm and 10 in the placebo. This sample will allow us to demonstrate the feasibility of conducting a full-scale randomised trial. This study is not powered to detect differences in treatment effect but will obtain estimates of an effect and its variance[30]. This sample size will allow us to estimate patient consent rate with a 95% confidence interval of ± 10% (based on 100 participants approached). The secondary outcome data will provide the required information for a sample size calculation for the primary end point of a subsequent trial[31]. Survival to discharge is above 96% for both study sites and hence it is likely that the ninety day follow data collection will be achieved for the majority of patients.

Recruitment rate

Over 275 patients per year will be eligible for study inclusion across the two study sites. With a ten month recruitment period, this requires a 13% recruitment rate. A recent interventional trial in liver transplantation at the RFH achieved a 53% recruitment rate[32]. Staff involvement and recruitment strategies will be similar in this study indicating this is a deliverable recruitment target.

Data collection

Data will be collected on trial-specific electronic case report forms (eCRFs) and anonymised data collated on a secure central trial database (MACRO, Elsevier, Amsterdam, Netherlands) on a server in UCL. Physical trial material will be stored locally at each study site in a secure setting. A trial specific data management standard operating procedure will contain details of the software to be used for the database, the process of database design, data entry, data quality checks, data queries, data security and database lock. At the end of the trial, all essential documentation will be archived securely for a minimum of 20 years from the declaration of the end of the trial.

All in-hospital data required for the study is part of routine medical care and as such is expected to have a high rate of completeness. Out-of-hospital data is similarly part of routine medical care. The completeness of study-specific PROM questionnaires performed before and after transplantation will be maximised through reminders by telephone and during clinic appointments.

Data will be analysed for completeness and accuracy through a check of 10% of patient records against the source data.

Third party data access will be assessed on a case-by-case basis by the Trial Management Group, approved by the Trial Steering Committee and limited specific anonymised data will be released, as appropriate. Patients will be consented for further analysis of anonymised data.

Statistical methods

A Statistical Analysis Plan will be approved by the Trial Steering Committee, prior to data analysis.

The following variables will be used to assess baseline comparability of the randomised groups;

- Study centre at which transplant performed,
- Age,
- Sex,

For peer review only

- The model for end-stage liver disease (MELD) score[33],
- The United Kingdom end-stage liver disease (UKELD) score[34],
- Aetiology of liver disease,
- The UK donor risk index[35],
- Organ graft undergoing normothermic machine perfusion,
- Presence of portal hypertension,
- Severity of pre-operative renal dysfunction (estimated glomerular filtration rate, eGFR),
- American Society of Anesthesiologists score,
- Pre-operative haemoglobin level,
- EuroQol-5D-5L questionnaire scores,
- Liver disease Quality of Life questionnaire score.

Patients will be analysed according to the groups to which there were randomised.

Primary outcomes

The primary outcome feasibility measures will be quantified using numbers (%). The degree of precision will be quantified using exact 95% confidence intervals.

Secondary outcomes

Numerical outcomes will be summarised using either mean (standard deviation) or medians (interquartile range) as appropriate. Binary and ordinal outcomes will be summarised using number (%). The degree of precision will be quantified using 95% confidence intervals. Differences between groups will be quantified using differences in means or proportions, as appropriate with corresponding 95% confidence intervals.

Since this is a feasibility study, no formal statistical tests are planned.

Sensitivity and other planned analyses

No sensitivity analyses are planned. There will be no interim analysis.

We will perform exploratory subgroup analyses for donor organ type (DCD and DBD), the presence of chronic kidney disease and known portal thrombosis (or if diagnosed at transplantation). We will also assess the impact of surgical approach (cross-clamp vs. any other approach), the use of machine perfusion vs. cold storage and hospital site.

Patient replacement

Patients who are randomised and allocated to a treatment arm but subsequently do not complete the trial will not be replaced.

Monitoring

Oversight committees

A Trial Management Group (TMG) and Trial Steering Committee (TSC) have been formed. The terms of reference and functions of these committees are provided in Appendix 4.

For peer review only

The TMG will include the Chief Investigators, co-investigators, trial staff, patient expert and study site Principle Investigators. The TMG will be responsible for overseeing the trial. The TMG will review recruitment figures, serious adverse events and substantial amendments to the protocol prior to submission to the Research Ethics Committee (REC).

Data monitoring

The TSC will represent a combined TSC and Data Monitoring Committee with expertise in performing and reporting clinical trials, statistical methods and clinical expertise for evaluation of post-transplant complications. The role of the TSC is to provide oversight for the trial and provide advice through its independent Chair to the Chief Investigator, the TMG, trial Sponsor, Funder, and host institutions on all aspects of the trial. The TSC will recommend any appropriate amendments/actions for the trial as necessary. The TSC is independent of the TMG, Funder and Sponsor and members have declared no competing interests.

Early stopping guidelines

The trial may be stopped before completion either on the recommendation of the TSC or the Sponsor and Chief Investigator. There will be no statistical stopping rules.

Adverse events

All adverse events will be recorded in the medical records in the first instance. All serious adverse events (SAEs) within 90 days of patient enrolment will be recorded in the eCRF with clinical symptoms, a brief description and date of the event. SAEs will be reported to the Sponsor within 24 hours. Where the event is unexpected and thought to be related to the intervention, this will be reported to the Health Research Authority within 15 days. Participants suffering an SAE will be followed up until clinical recovery is complete and laboratory results have returned to expected values, or until the event has stabilised. Follow-up will continue after completion of trial follow-up if necessary. For the purpose of analysis, SAEs prior to discharge, within 30 days and within 90 days of enrolment will be analysed and published.

The sponsor will notify the REC and MHRA of all suspected unexpected serious adverse reactions (SUSARs) within 15 days. SUSARs that are fatal or life-threatening will be notified to the MHRA and REC within 7 days of the sponsor learning of them.

Auditing

Given the short duration of this study no formal audit is planned. All documentation will be collected and recorded in keeping with Good Clinical Practice guidelines and will be available for external review or auditing if required.

ETHICS AND DISSEMINATION

Research ethics approval

This study has been given approval by the East Midlands - Leicester South Research Ethics Committee (IRAS reference: 278918, REC reference: 21/EM/0076) and the Health Research Authority. This feasibility study does not require MHRA approval.

For peer review only

Protocol amendments

Substantial protocol amendments will be reviewed by the TMG, TSC, and Sponsor prior to REC and Health Research Authority submission.

Any changes from this protocol paper will be highlighted in the study outcome publication.

Confidentiality

All investigators and trial site staff will comply with the requirements of General Data Protection Regulation with regard to the collection, storage, processing and disclosure of personal information and will uphold the Data Protection Act's core principles. All patient records will remain strictly confidential at all times. All patients recruited to the study will be allocated a unique trial identification number that will be used on all study-specific correspondence. All study information will be held securely on paper and electronically on eCRFs. Any patient information which leaves the hospital will only contain the trial identifier. Patient identifiable details will not be passed to anyone outside the research team or the study Sponsor.

Declaration of interests

The Chief and Principal Investigators and members of the TMG have declared that they have no financial or other competing interests.

Ancillary and post-trial care

The Sponsor holds insurance against claims from participants for injury caused by their participation in the trial. Each study site hospital will provide negligence insurance cover for harm caused by their employees. All ongoing care for the treatment of adverse events will be provided by the recruiting hospital.

Dissemination policy

A publication policy will be written and agreed by the TMG prior to trial data analysis. This will be agreed with the TSC. The main study outcomes will be submitted to an open-access journal.

Patient and public involvement

Interviews and engagement with liver transplantation patients and their families has been a key motivator behind this study and a source of knowledge to identify outcomes that have been actively promoted in study design. A reduction in perioperative complications, especially haemorrhage, was identified by patients as a main research priority as well as physical and psychosocial wellbeing and quality of life after surgery. The PROM questionnaires were assessed during patient and family interviews to ascertain their accessibility, value and feasibility. The PIS has been reviewed with pre-operative patients and family to obtain feedback on its clarity, readability and content. A transplant recipient patient expert is a co-applicant to this grant and will be an active member of the TMG. Our patient expert will assist us in providing effective communication with patients and public with a patient-centred viewpoint.

Author contribution

For peer review only

Writing and approving manuscript - JF, MS, DM, BD, BS, GA, NW.

Reviewing and approving manuscript - JF, MS, DM, BD, BS, GA, NW.

Funding

This project is funded by the National Institute for Health Research (NIHR) under its Research for Patient Benefit (RfPB) Programme (Grant Reference Number PB-PG-0817-20023). The views expressed are those of the author(s) and not necessarily those of the NIHR or the Department of Health and Social Care.

Competing interests statement

All authors declare that they have no financial or other competing interests.

For peer review only

REFERENCES

1. NHSBT. Annual Report on Liver Transplantation (2019-2020). online] 2020. Accessed 20/04/2021.
2. Rana A, Petrowsky H, Hong JC, et al. Blood transfusion requirement during liver transplantation is an important risk factor for mortality. J Am Coll Surg 2013;216:902-907.
3. de Boer MT, Christensen MC, Asmussen M, et al. The impact of intraoperative transfusion of platelets and red blood cells on survival after liver transplantation. Anesth Analg 2008;106:32-44, table of contents.
4. Reichert B, Kaltenborn A, Becker T, Schiffer M, Klempnauer J, Schrem H. Massive blood transfusion after the first cut in liver transplantation predicts renal outcome and survival. Langenbeck's archives of surgery 2014;399:429-440.
5. D'Amico G, Pagliaro L, Bosch J. Pharmacological Treatment of Portal Hypertension: An Evidence-Based Approach. Semin Liver Dis 1999;19:475-505.
6. Chen X, Ding X, Shen B, et al. Incidence and outcomes of acute kidney injury in patients with hepatocellular carcinoma after liver transplantation. J Cancer Res Clin Oncol 2017.
7. Rahman S, Davidson B, Mallett S. Early acute kidney injury after liver transplantation: Predisposing factors and clinical implications. World J Hepatol 2017.
8. Ayhan A, Ersoy Z, Ulas A, Zeyneloglu P, Pirat A, Haberal M. Incidence and Patient Outcomes in Renal Replacement Therapy After Orthotopic Liver Transplant. Exp Clin Transplant 2017;15:258-260.
9. Zhu M, Li Y, Xia Q, et al. Strong impact of acute kidney injury on survival after liver transplantation. Transplantation proceedings 2010;42:3634-3638.
10. Lima EQ, Zanetta DM, Castro I, et al. Risk factors for development of acute renal failure after liver transplantation. Ren Fail 2003;25:553-560.
11. Yalavarthy R, Edelstein CL, Teitelbaum I. Acute renal failure and chronic kidney disease following liver transplantation. Hemodialysis international International Symposium on Home Hemodialysis 2007;11 Suppl 3:S7-12.
12. Mountokalakis T, Kallivretakis N, Mayopoulou-Symvoulidou D, Karvountzis G, Tolis G. Enhancement of renal function by a long-acting somatostatin analogue in patients with decompensated cirrhosis. Nephrol Dial Transplant 1988;3:604-607.
13. Kaffy F, Borderie C, Chagneau C, et al. Octreotide in the treatment of the hepatorenal syndrome in cirrhotic patients. Journal of hepatology 1999;30:174.
14. Sun H, Zou S, Candiotti KA, et al. Octreotide Attenuates Acute Kidney Injury after Hepatic Ischemia and Reperfusion by Enhancing Autophagy. Sci Rep 2017;7:42701.
15. Sahmeddini MA, Amini A, Naderi N. The effect of octreotide on urine output during orthotopic liver transplantation and early postoperative renal function; a randomized, double-blind, placebo-controlled trial. Hepat Mon 2013;13:e12787.
16. Cacciarelli TV, Keeffe EB, Moore DH, et al. Effect of intraoperative blood transfusion on patient outcome in hepatic transplantation. Arch Surg 1999;134:25-29.
17. Boin IF, Leonardi MI, Luzo AC, Cardoso AR, Caruy CA, Leonardi LS. Intraoperative massive transfusion decreases survival after liver transplantation. Transplantation proceedings 2008;40:789-791.
18. Xia VW, Du B, Braunfeld M, et al. Preoperative characteristics and intraoperative transfusion and vasopressor requirements in patients with low vs. high MELD scores. Liver transplantation : official publication of the American Association for the Study of Liver Diseases and the International Liver Transplantation Society 2006;12:614-620.
19. Garcia-Pagan JC, Escorsell A, Moitinho E, Bosch J. Influence of pharmacological agents on portal hemodynamics: basis for its use in the treatment of portal hypertension. Semin Liver Dis 1999;19:427-438.

For peer review only

20. Ottesen LH, Aagaard NK, Kiszka-Kanowitz M, et al. Effects of a long-acting formulation of octreotide on renal function and renal sodium handling in cirrhotic patients with portal hypertension: a randomized, double-blind, controlled trial. Hepatology 2001;34:471-477.
21. Velez JC, Nietert PJ. Therapeutic response to vasoconstrictors in hepatorenal syndrome parallels increase in mean arterial pressure: a pooled analysis of clinical trials. American journal of kidney diseases : the official journal of the National Kidney Foundation 2011;58:928-938.
22. Mizota T, Hamada M, Matsukawa S, Seo H, Tanaka T, Segawa H. Relationship Between Intraoperative Hypotension and Acute Kidney Injury After Living Donor Liver Transplantation: A Retrospective Analysis. J Cardiothorac Vasc Anesth 2017;31:582-589.
23. Hilmi IA, Damian D, Al-Khafaji A, et al. Acute kidney injury after orthotopic liver transplantation using living donor versus deceased donor grafts: A propensity score-matched analysis. Liver transplantation : official publication of the American Association for the Study of Liver Diseases and the International Liver Transplantation Society 2015;21:1179-1185.
24. Byram SW, Gupta RA, Ander M, Edelstein S, Andreatta B. Effects of Continuous Octreotide Infusion on Intraoperative Transfusion Requirements During Orthotopic Liver Transplantation. Transplantation proceedings 2015;47:2712-2714.
25. Troisi R, Croo A, Codarin E, et al. Somatostatin (Eumedica®) Infusion Allows Modulating Graft Flows and Gradients in Liver Transplantation. Results of a Randomized, Double Blind, Placebo Controlled Trial [abstract]. In: American Transplant Congress; 2015: Am J Transplant, 2015.
26. Chan AW, Tetzlaff JM, Altman DG, et al. SPIRIT 2013 statement: defining standard protocol items for clinical trials. Annals of internal medicine 2013;158:200-207.
27. Moher D, Hopewell S, Schulz KF, et al. CONSORT 2010 explanation and elaboration: updated guidelines for reporting parallel group randomised trials. BMJ 2010;340:c869.
28. Gralnek IM, Hays RD, Kilbourne A, et al. Development and evaluation of the Liver Disease Quality of Life instrument in persons with advanced, chronic liver disease--the LDQOL 1.0. The American journal of gastroenterology 2000;95:3552-3565.
29. EuroQol--a new facility for the measurement of health-related quality of life. Health Policy 1990;16:199-208.
30. Julious SA. Sample size of 12 per group rule of thumb for a pilot study. Pharmaceutical Statistics 2005;4:287-291.
31. Sim J, Lewis M. The size of a pilot study for a clinical trial should be calculated in relation to considerations of precision and efficiency. Journal of clinical epidemiology 2012;65:301-308.
32. Martin D. Optimising the cardiovascular system following liver transplantation surgery. Study code ISRCTN10329248 [online]. Available at: http://www.isrctn.com/ISRCTN10329248.
33. Kamath PS, Kim WR. The model for end-stage liver disease (MELD). Hepatology 2007;45:797-805.
34. Barber K, Madden S, Allen J, Collett D, Neuberger J, Gimson A. Elective liver transplant list mortality: development of a United Kingdom end-stage liver disease score. Transplantation 2011;92:469-476.
35. Collett D, Friend PJ, Watson CJ. Factors Associated With Short- and Long-term Liver Graft Survival in the United Kingdom: Development of a UK Donor Liver Index. Transplantation 2017;101:786-792.

For peer review only

Table 1: World Health Organization Trial Registration Data Set

**Data category**	**Information**
Primary registry and trial identifying number	ClinicalTrials.gov<br>NCT04941911
Date of registration in primary registry	June 28, 2021
Source(s) of monetary or material support	National Institute of Health Research
Primary sponsor	University College London, United Kingdom
Contact for public queries	MS [michaelspiro@nhs.net]
Contact for scientific queries	MS [michaelspiro@nhs.net]
Public title	A Feasibility Study of Octreotide Infusion During Liver Transplant
Scientific title	A Double-blind Randomised Placebo-controlled Feasibility Study to Assess the Impact of Octreotide Infusion During Liver Transplantation on Post-operative Renal Failure
Countries of recruitment	United Kingdom
Health condition(s) or problem(s) studied	Liver transplantation
Intervention(s)	Active comparator: Intravenous Octreotide acetate infusion, 100mcg bolus with a subsequent infusion of 100mcg per hour during surgery
	Placebo comparator: Sodium chloride 0.9% w/v
Key inclusion and exclusion criteria	Ages eligible for study: ≥18 years<br>Sexes eligible for study: both<br>Accepts healthy volunteers: no
	Inclusion criteria: adults aged 18 years and over undergoing primary liver transplantation of a whole or partial liver graft from a cardiac or brain dead donor
	Exclusion criteria: previous solid organ transplant, acute liver

For peer review only

Data category	Information
	failure, fulminant hepatic failure, patients receiving a living donor liver graft, patients currently admitted to ICU prior to transplantation, requirement of haemodialysis or CVVHF pre-operatively, known allergy or adverse reaction to octreotide, pre-operative decision to use intra-operative CVVHF, a positive pregnancy test.
Study type	Interventional
	Allocation: patients will be randomised in a 2:1 ratio to either octreotide or placebo groups. Stratified randomisation of patients by source of liver graft (brain death or cardiac death).<br>Masking: quadruple (Participant, Care Provider, Investigator, Outcomes Assessor)
	Primary purpose: feasibility
	Phase II
Date of first enrolment	July 2021
Target sample size	30
Recruitment status	Pending recruitment
Primary outcome(s)	Trial feasibility
Key secondary outcomes	Incidence of acute kidney injury<br>Post-operative incidence of a new requirement for renal replacement therapy<br>Incidence of new chronic kidney disease or deterioration of chronic kidney disease<br>Incidence of early allograft dysfunction<br>Patient mortality<br>Intra-operative red blood cell salvage<br>Volume of packed red blood cell transfusion administered

For peer review only

Data category	Information
	Incidence of adverse events secondary to study drug infusion<br><br>PROMs data

For peer review only

Appendix 1: Study Flowchart

Figure legend: Study flowchart showing timing of major study phases and events.

Appendix 2: Informed Consent Form

Appendix 3: Patient Information Sheet

For peer review only

Appendix 4: TMG and TSC terms of reference and functions

Responsibilities of the combined Trial Steering Committee and Data Monitoring Committee

To act as the oversight body for the trial on behalf of the Sponsor and Funder.

The role of the TSC is to provide oversight for the trial and provide advice through its independent Chair to the Chief Investigator, the TMG, trial Sponsor, Funder, and host institution on all aspects of the trial. The rights, safety and well-being of the trial participants are the most important consideration and should prevail over the interests of science and society.

Functions of the TSC include:

- Provide expert oversight of the trial
- Maintain confidentiality of all trial information not already in the public domain
- Review regular progress reports from the trial team
- Monitor recruitment rates and review strategies proposed to deal with any recruitment problems
- Make decisions regarding continuation or termination of the trial
- Assess the impact and relevance of any accumulating external evidence
- Monitor quality of the data and review strategies proposed to improve data collection
- Monitor follow-up rates and review strategies proposed to deal with problems
- Monitor sites that are deviating from the protocol
- Review any proposed amendments to the protocol
- Review proposals by the TMG concerning any change to the design of the trial, including additional sub-studies
- Oversee the timely reporting to trial results
- Review the statistical analysis plan
- Review the publication policy
- Review the main trial manuscript
- Review any abstracts and presentations of results during the trial
- Approve external or early internal requests for release of data, subsets of data or samples including clinical data.

For peer review only

Responsibilities of the Trial Management Group

- Input into and comment on the protocol and data collection methods
- Promote the trial
- Develop strategies to encourage recruitment and address any issues with recruitment at participating sites
- Be involved in the day-to-day running of the trial by supporting the CI and trial coordinators
- Provide clinical or other expert guidance to the trial coordinators and participating sites on trial- based matters such as clinical and practical queries and interpretation of information recorded on eCRFs or other data collection tools
- Maintain confidentiality of any trial information that is not in the public domain
- Respond to trial correspondence and any questions in a timely fashion
- Review data collection at sites
- Input into the monitoring and classification of SAEs where clinically appropriate
- Input into the meetings of the TSC, if appropriate
- Provide responses to any issues or concerns raised by TSC
- Be aware of accumulating external evidence and assess its impact and relevance
- Input into the development of the Statistical Analysis Plan where appropriate
- Input into the interpretation and trial report writing
- Report to the TSC regarding trial progress

For peer review only

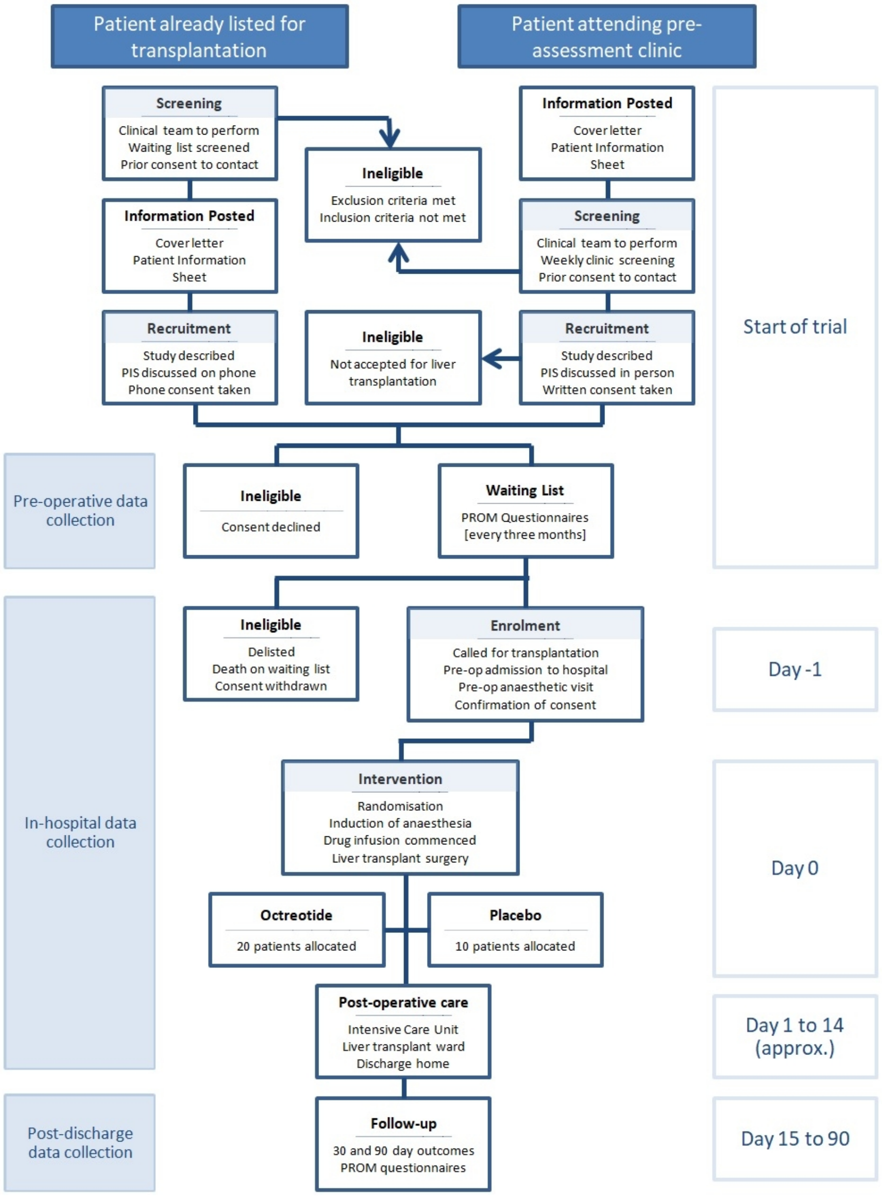

Study Flowchart

248x335mm (96 x 96 DPI)

Study Number: IRAS 278918
Sponsor Reference number 17/0508
Centre Number:
Patient Identification Number/Subject Number for this trial:

INFORMED CONSENT FORM (ICF)

In-Person Consent

Project Title Assessing the Impact of Octreotide Infusion during Liver Transplantation

For peer review only

Confirmation of consent to be performed on admission for transplantation
Points one to seven to be confirmed
Patient confirmation signature overleaf

Please initial box

1. I confirm that I have read and understand the information sheet dated 15.01.2021 (version 1.0) for the above study. I have had the opportunity to consider the information, ask questions and have had these answered satisfactorily. ☐

2. I understand that my participation is voluntary and that I am free to withdraw at any time without giving any reason, without my medical care or legal rights being affected. ☐

3. I understand that relevant sections of my medical notes and data collected during the study, may be looked at by individuals from the sponsor of the trial (University College London) and responsible persons authorised by the sponsor, from regulatory authorities or from the NHS Trust, where it is relevant to my taking part in this research. I give permission for these individuals to have access to my records. ☐

4. I agree to my GP being informed of my participation in the study. ☐

5. I consent to the collection, processing, reporting, storage and transfer within and outside Europe of my anonymised data for healthcare and/or medical research purposes in relation to this research. I understand that I will not be directly identifiable except to the study doctor and his/her study team. ☐

6. I agree to take part in the above study. ☐

7. After the entire study has completed I would like to receive a letter indicating which medication (octreotide or placebo) I received. [Optional]. ☐

For peer review only - http://bmjopen.bmj.com/site/about/guidelines.xhtml

Name of patient | Date | Signature

Name of person taking consent | Date | Signature

Name of Chief Investigator | Date | Signature
(if different to the person taking consent)

When completed: 1 for participant; 1 (original) for researcher site file; 1 to be kept in medical notes. Please file a copy of the GP letter in the medical records and write a note in the medical record stating when the patient consented and date when the GP letter was sent.

Confirmation of consent on admission for transplantation

Name of patient | Date | Signature

Name of person taking consent | Date | Signature

For peer review only - http://bmjopen.bmj.com/site/about/guidelines.xhtml

**Full title of trial**	A double-blind randomised placebo-controlled feasibility study to assess the impact of octreotide infusion during liver transplantation on post-operative renal failure.
**Short title**	A feasibility study of octreotide infusion during liver transplant.

Patient Information Sheet

Study title: A feasibility study of octreotide infusion during liver transplant.

Protocol Reference Number: Sponsor Ref number: 17/0508

We would like to invite you to take part in a research study. Before you decide we would like you to understand why the research is being done and what it would involve for you. One of our team will go through the information sheet with you when you attend hospital for your assessment and answer any questions you have. We would suggest this should take about ten minutes. Talk to others about the study if you wish.

Part 1 tells you the purpose of this study and what will happen to you if you take part. Part 2 gives you more detailed information about the conduct of the study.

Ask us if there is anything that is not clear. Take time to decide whether or not you wish to take part.

Part 1

What is the purpose of the study?

Liver transplant surgery can lead to effects on the kidneys requiring medical support including kidney dialysis. The surgery also has a risk of bleeding and the need for blood transfusion. These complications can slow down recovery after the operation and decrease the chances of the new liver working.

We are investigating a drug called octreotide that is very similar to a natural hormone present in the human body called somatostatin. Octreotide is already used regularly to treat kidney failure in liver disease. We already have evidence that octreotide improves how well the kidney creates urine during and after liver transplant surgery. There is also some evidence that octreotide improves patients' blood pressure in liver disease and during liver transplant surgery and this helps improve kidney function. One published study showed that octreotide might also reduce the amount of blood transfusion required during transplant surgery.

For peer review only - http://bmjopen.bmj.com/site/about/guidelines.xhtml

We are running a research study to find out whether octreotide can improve kidney function during and after transplant surgery and leads to fewer patients needing to have kidney support. We will also look at whether octreotide helps stabilise blood pressure during surgery, reduce bleeding and the need for blood transfusion.

Octreotide is used regularly in some hospitals during liver transplant surgery but it is not clear whether it is beneficial. The quality of research that has been done on octreotide in liver transplant surgery is of poor quality. This research study aims to show if octreotide does have a benefit or not and whether this leads to long-term improvements in patients' health including their survival, the function of the new liver and quality of life.

Why have I been invited?

You have been invited to take part in this clinical trial because you have been referred to either the Royal Free or Queen Elizabeth Hospital for a liver transplant and meet all of the research study criteria for patients to be included. We foresee around thirty patients being involved in this research project over the next two years.

The research team running this clinical trial includes specialists from the liver transplant team, anaesthesia and intensive care as well as liver and kidney specialists.

Is there anything that would stop me from taking part?

If you are undergoing surgery to transplant any organs other than a liver transplant at the same time or if you have previously received any organ transplant, then you would not be able to take part in this study. You would also not be able to take part if you are receiving a 'living donor' liver organ, are suffering from severe liver failure called fulminant hepatic failure or if you have a known allergy to octreotide.

Do I have to take part?

It is up to you to decide to join the study. We will describe the study and go through this information sheet. If you agree to take part, we will then ask you to sign a consent form. You are free to withdraw at any time without giving a reason. Whether or not you are involved in this study, or if you decide to withdraw from the study at any time, will not affect the care you receive. Your involvement in this study will not have any effect on the chances of you receiving a liver transplant.

What will happen to me if I take part?

If you decide to take part then we will ask you to sign an informed written consent form prior to the study procedure.

All of the study takes place in the liver transplant operating theatre while you are under general anaesthetic at the Royal Free or Queen Elizabeth Hospital.

For peer review only - http://bmjopen.bmj.com/site/about/guidelines.xhtml

Your experience of surgery and your care both before and after the transplant procedure will be identical to patients who are not taking part in the study.

In order to find out whether or not octreotide provides a benefit to patients we need to compare patient outcomes between those who have and have not had octreotide. To make the study as effective as possible we will compare the octreotide drug to a placebo. A placebo is a dummy treatment that looks like the real thing but is not. It contains no active ingredient. Patients who agree to be part of this study will receive either an infusion of octreotide or an infusion of an inactive drug (a saline solution).

To make the study as high a quality of research as is possible it will be performed in a 'double blind' fashion. In a 'blind trial' you will not know which treatment group you are in. As this trial is a 'double blind trial', neither you nor your doctor will know in which treatment group you are (although, if your doctor needs to find out he/she can do so).

Sometimes we don't know which way of treating patients is best. To find out, we need to compare different treatments. We put people into groups and give each group a different treatment. The results are compared to see if one is better. To try to make sure the groups are the same to start with, each patient is put into a group by chance (randomly). You have a two-in-three chance of your receiving the active drug (octreotide) in this study, and a one-in-three chance of the inactive drug.

This study is known as a 'feasibility study'. The idea of this type of research study is to find out whether a much larger trial would be possible and how it should be designed. The design of the study is identical to a larger trial but has a smaller number of patients in it.

Neither you nor the doctors looking after you will be aware of if you receive the active drug or not. We will monitor you very closely and record your data throughout your transplant and whilst on intensive care and the ward. The infusion will be started after you are asleep for your liver transplant and be stopped before you are transferred to the intensive care unit.

You will have received this Patient Information Sheet either by post or during your visit to The Royal Free London or Queen Elizabeth Hospital Birmingham. A member of the research team will contact you by phone if you are at home, or visit you whilst you are in the hospital to fully explain this study and offer you the opportunity to be involved. If you wish to take part we will complete a consent form with you when you attend the hospital on the day of your liver transplant.

Your involvement in the research project will be from the time you consent to being involved until ninety days after your liver transplant surgery as we will collect information during your recovery from the transplant procedure.

For peer review only - http://bmjopen.bmj.com/site/about/guidelines.xhtml

When you receive this Patient information leaflet, you will discuss the information with family, friends and/or GP. If interested then contact study team, who will discuss the study with you and you can register an expression of interest and if eligible you will be consented to d be involved in study. If you do not proceed to transplant over the study period you cannot be included in the trial.

The study will involve a small number of questionnaires but will not require you to attend any additional clinics or hospital appointments. These include:

- The completion of an written informed consent form,
- The completion of two quality of life questionnaires (taking roughly thirty minutes) during the pre-operative assessment,
- The completion of two quality of life questionnaires around three months after surgery. These will be done at your routine follow-up appointment and take roughly thirty minutes.

Patients who agree to take part in this study will receive all normal care and treatments for their liver transplant, no treatments will be withheld as part of this study. There is no video or audiotaping or photography involved in this study.

Expenses and payments

There are no expenses or payments involved in this study.

What are the possible disadvantages and risks of taking part?

Octreotide is an artificial version of a hormone that the body produces constantly to help regulate the gut and digestion. Octreotide is used routinely in the NHS for treatment of kidney failure and bleeding in liver disease. Some hospitals use octreotide routinely during liver transplant surgery and feel it provides benefits. Octreotide is a used quite commonly in patients who have liver disease. It is generally regarded as a very safe medication.

However, there are risks with all medical treatments. Effects that have been found from the use of octreotide are:

- Changes in blood sugar levels
- A slowing down in the heart rate
- Changes in heart rhythm

During and after liver transplant surgery the heart rate and rhythm is monitored constantly and if there are any concerns this can be treated. Your blood sugar levels are checked approximately hourly throughout surgery and at a similar frequency

For peer review only - http://bmjopen.bmj.com/site/about/guidelines.xhtml

after surgery on the intensive care unit. If there are concerns regarding your blood sugar levels these are treated routinely.

If at any point during your surgery there is a concern that the medication is causing any problems that cannot be easily treated then the trial medication will be stopped.

There are no risks associated with the placebo infusion.

Before participating you should consider if this will affect any insurance you have and seek advice if necessary.

What are the effects of any treatment received when taking part?

Octreotide has been used in the NHS for a significant period of time and the effects of the medication are well understood.

Octreotide can cause low blood sugar levels in patients with diabetes and increase the effect of drugs taken to control blood sugar levels. Octreotide can also increase blood sugar levels in patients with and without diabetes. All patients in this study will have regular monitoring of blood sugar concentrations throughout their surgery and afterwards on the intensive care unit. All medicines affecting blood sugar levels are stopped routinely before surgery and blood sugar levels are controlled as part of routine care after surgery.

Octreotide may increase the effect of some medications that slow the heart or adjust the heart rhythm. During surgery and while on the intensive care unit the heart rate and rhythm of all patients is monitored continuously as part of routine care and kept in a safe range.

Octreotide can slow down how the body breaks down the medication codeine and this may reduce the pain relief provided by this medication. Codeine is not used as part of routine pain relief for patients who have undergone liver transplant surgery.

Octreotide may decrease the levels of a drug used to suppress the immune system called cyclosporine. Cyclosporin used to be used to reduce the change that the immune system would reject the new liver that had been transplanted. Cyclosporin is no longer used to suppress the immune system in liver transplantation.

There are no effects associated with the placebo infusion.

Anaesthetists, intensive care doctors, and other medical practitioners involved in the care of patients involved in this study will be aware of these possible effects of octreotide. Members of the research and pharmacy team who care for patients on this research study will monitor patients' medications to ensure the chance of effects is made as small as possible.

For peer review only - http://bmjopen.bmj.com/site/about/guidelines.xhtml

We would expect the body to have removed all the octreotide medication within 48 hours after surgery. As such, there will be no concerns regarding octreotide after you have left hospital following your liver transplant surgery.

There are no recognized risks from octreotide to pregnant women. A pregnancy test is required for all women who are at are at risk of pregnancy before they can undergo liver transplant surgery. A positive pregnancy test would prevent you from taking part in this study. If you take part in this study there are no restrictions on you becoming pregnant following your liver transplant surgery, other than those given to you by your clinical care team.

What are the possible benefits of taking part?

We cannot promise the study will help you but the information we get from this study will help improve the treatment of people who are receiving future liver transplants.

Octreotide use during liver transplant surgery is a standard part of care in some of the world's top transplant institutes. The evidence we have so far suggests that octreotide infusions may reduce the chances of kidney failure after surgery, improve blood pressure, reduce bleeding and the need for blood transfusion. As such, if you receive the octreotide infusion your care may benefit, although we cannot be certain.

What happens when the research study stops?

Only a single treatment of octreotide or placebo will be given in this study. There is no evidence to suggest that further doses of octreotide after surgery would be of any benefit.

Your medical care after liver transplant surgery will be identical to all other patients who are not part of this research study. As such, there will be no changes after the research study stops.

It will not be possible to tell patients which medication they were given during the research study.

Once the study has been completed the data will be analysed and this may suggest that we should use Octreotide routinely. If so this could be introduced for future transplant patients. It is more likely that the data will indicate that a larger study is required to demonstrate whether Octreotide is beneficial in this situation.

What if there is a problem?

If you seem to have an adverse reaction to this medication, we will immediately stop the infusion of the trial medication and treat the complication if needed. We will document what has happened and inform you of what has happened.

For peer review only - http://bmjopen.bmj.com/site/about/guidelines.xhtml

Any complaint about the way you have been dealt with during the clinical trial or any possible harm you might suffer will be addressed. The detailed information concerning this is given in Part 2 of this information sheet. If you have any concerns or complaints you should contact your study doctor in the first instance.

Will my taking part in the study be kept confidential?

Yes. We will follow ethical and legal practice and all information about you will be handled in confidence. The details are included in Part 2.

For peer review only

For peer review only - http://bmjopen.bmj.com/site/about/guidelines.xhtml

Contact Details

Your Doctor

Queen Elizabeth Hospital, Birmingham

Consultant Anaesthetist

Royal Free Hospital, London

Consultant Anaesthetist and Intensivist

Your Research Nurse

Insert local contact details

Name

Tel. Number:

This completes Part 1 of the Information Sheet.

If the information in Part 1 has interested you and you are considering participation, please read the additional information in Part 2 before making any decision.

For peer review only - http://bmjopen.bmj.com/site/about/guidelines.xhtml
For peer review only

Part 2

What if relevant new information becomes available?

Sometimes we get new information about the treatment being studied. If this happens, we will tell you about it and discuss whether you want to or should continue in the study. If you decide not to carry on there will be no changes to your normal care before and after your transplant surgery. If you decide to continue in the study we will ask you to sign an updated consent form.

If the study is stopped for any other reason, we will tell you why and ensure your continuing care is unaffected.

What will happen if I don't want to carry on with the study?

You can withdraw from the study at any time without giving a reason, either before or after your liver transplant surgery. It will not affect your future care in any way.

If you choose to withdraw from the study before your liver transplant surgery then you will not be given the trial medication and any data that can be identified as yours can be destroyed if you wish.

If you choose to withdraw from the study after your liver transplant surgery then we would continue to collect and inspect data regarding drug safety and effects. We would also request that your data is used for the study analysis. However, we would not collect any other data and data not relating to this, for instance to do with your quality of life, would be destroyed if you wished. This is because we would have duty of care to you to ensure that you were safe having received the study medication.

What if there is a problem?

If you have a concern about any aspect of this study you should speak directly to the study organiser or a member of the research team. The anaesthetic and intensive care team involved in your care will be able to contact them for you directly. If you wish to complain about any aspect of the way in which you have been approached or treated during the course of this study, you can contact the study doctors through their contact details, below.

You may also wish to contact the Royal Free or Queen Elizabeth Hospital Patient Advice and Liaison Service on 020 7472 6446 (Royal Free) or 0121 424 0808 (Queen Elizabeth Hospital). They offer offers support, information and assistance to patients, relatives and visitors about medical services and hospital care.

Every care will be taken in the course of this clinical trial. However, in the unlikely event that you are injured by taking part, compensation may be available.

For peer review only - http://bmjopen.bmj.com/site/about/guidelines.xhtml

If you suspect that the injury is the result of the Sponsor's (University College London) or the hospital's negligence, then you may be able to claim compensation. After discussing with your study doctor, please make the claim in writing to the Dr. Michael Spiro who is the Chief Investigator for the clinical trial and is based at The Royal Free Hospital, Department of Anaesthesia. The Chief Investigator will then pass the claim to the Sponsor's Insurers, via the Sponsor's office. You may have to bear the costs of the legal action initially, and you should consult a lawyer about this.

Participants may also be able to claim compensation for injury caused by participation in this clinical trial without the need to prove negligence on the part of University College London or another party. You should discuss this possibility with your study doctor in the same way as above.

Regardless of this, if you wish to complain, or have any concerns about any aspect of the way you have been approached or treated by members of staff or about any effects (adverse events) you may have experienced due to your participation in the clinical trial, the normal National Health Service complaints mechanisms are available to you. Please ask your study doctor if you would like more information on this. Details can also be obtained from the Department of Health website: http://www.dh.gov.uk.

Will my taking part in this study be kept confidential?

If you consent to take part in this study, the records obtained while you are in this study as well as related health records will remain strictly confidential at all times. The information will be held securely on paper and electronically at your treating hospital and the main hospital site managing this research (The Royal Free Hospital, London and University Hospitals Birmingham NHS Foundation Trust) under the provisions of the 1998 Data Protection Act. Your name will not be passed to anyone else out the research team or the Sponsor (UCL), who is not involved in the trial. You will be allocated a trial number, which will be used as a code to identify you on all trial forms. Any information about you which leaves the hospital/surgery will have your name and address removed so that you cannot be recognised.

Your records will be available to people authorised to work on the trial but may also need to be made available to people authorised by the Sponsor, which is the organisation responsible for ensuring that the study is carried out correctly. By signing the consent form you agree to this access for the current study and any further research that may be conducted in relation to it, even if you withdraw from the current study. Data collected during this phase of the study will be included in a larger follow-on study performed in an identical fashion.

The information collected about you may also be shown to authorised people from the UK Regulatory Authority (the Medicines and Healthcare Products Regulatory Authority); this is to ensure that the study is carried out to the highest possible scientific standards. All will have a duty of confidentiality to you as a research participant.

For peer review only - http://bmjopen.bmj.com/site/about/guidelines.xhtml

If you withdraw consent from further study treatment, unless you object, your data and samples will remain on file and will be included in the final study analysis.

In line with the regulations, at the end of the study your data will be securely archived for a minimum of 20 years. Arrangements for confidential destruction will then be made.

Anonymised Data collected during the study may be transferred for the purpose of analysis to associated researchers within/out the European Economic Area. Some countries outside of Europe may not have laws which protect your privacy to the same extent as the Data Protection Act in the UK or European Law. The Sponsor of the trial will take all reasonable steps to protect your privacy.

Will my GP be informed of my involvement?

With your permission, your GP, and other doctors who may be treating you, will be notified that you are taking part in this study.

What will happen to any samples I give?

No samples will be taken.

Will any genetic tests be done?

No genetic tests will be performed.

What will happen to the results of the research study?

The results of the study will be available after it finishes and will usually be published in a medical journal or be presented at a scientific conference. The data will be anonymous and none of the patients involved in the trial will be identified in any report or publication.

Should you wish to see the results, or the publication, please ask your study doctor. We will send all interested patients a copy of the study results and will arrange a presentation of the research findings to the participants and the wider liver transplant community.

Who is organising and funding the research?

The research is being funded by the National Institute for Health Research as part of their Research for Patient Benefit programme. The doctors involved in setting up and running this research project are not being paid to do so. The doctors and wider research team do not have any conflict of interests in this study and no companies or members of the pharmaceutical industry are involved in any way.

Who has reviewed the study?

For peer review only - http://bmjopen.bmj.com/site/about/guidelines.xhtml

All research in the NHS is looked at by independent group of people, called a Research Ethics Committee, to protect your interests. This study has been reviewed and given favourable opinion by East Midlands - Leicester South Research Ethics Committee.

Further information and contact details

You are encouraged to ask any questions you wish, before, during or after your treatment. If you have any questions about the study, please speak to your study nurse or doctor, who will be able to provide you with up to date information about the drug(s)/procedure(s) involved. If you wish to read the research on which this study is based, please ask your study nurse or doctor. If you require any further information or have any concerns while taking part in the study, please contact one of the following people:

Your Doctor

Queen Elizabeth Hospital, Birmingham

Consultant Anaesthetist

Royal Free Hospital, London

Consultant Anaesthetist and Intensivist

Your Research Nurse

Name

Tel. Number:

Alternatively, if you or your relatives have any questions about this study you may wish to contact one of the following organisations that are independent of the hospital at which you are being treated:

If you decide you would like to take part then please read and sign the consent form. You will be given a copy of this information sheet and the consent form to keep. A copy of the consent form will be filed in your patient notes, one will be filed with the study records and one may be sent to the Research Sponsor.

You can have more time to think this over if you are at all unsure.

For peer review only - http://bmjopen.bmj.com/site/about/guidelines.xhtml

Thank you for taking the time to read this information sheet and to consider this study.

For peer review only - http://bmjopen.bmj.com/site/about/guidelines.xhtml

Reporting checklist for protocol of a clinical trial

		Reporting Item	Page Number
**Administrative information**			
Title	#1	Descriptive title identifying the study design, population, interventions, and, if applicable, trial acronym	1
Trial registration	#2a	Trial identifier and registry name. If not yet registered, name of intended registry	2
Trial registration: data set	#2b	All items from the World Health Organization Trial Registration Data Set	16-19
Protocol version	#3	Date and version identifier	2
Funding	#4	Sources and types of financial, material, and other support	2
Roles and responsibilities: contributorship	#5a	Names, affiliations, and roles of protocol contributors	1
Roles and responsibilities: sponsor contact information	#5b	Name and contact information for the trial sponsor	3

For peer review only

Roles and responsibilities: sponsor and funder	#5c	Role of study sponsor and funders, if any, in study design; collection, management, analysis, and interpretation of data; writing of the report; and the decision to submit the report for publication, including whether they will have ultimate authority over any of these activities	3
Roles and responsibilities: committees	#5d	Composition, roles, and responsibilities of the coordinating centre, steering committee, endpoint adjudication committee, data management team, and other individuals or groups overseeing the trial, if applicable (see Item 21a for data monitoring committee)	3
**Introduction**			
Background and rationale	#6a	Description of research question and justification for undertaking the trial, including summary of relevant studies (published and unpublished) examining benefits and harms for each intervention	4-5
Background and rationale: choice of comparators	#6b	Explanation for choice of comparators	4-5
Objectives	#7	Specific objectives or hypotheses	5
Trial design	#8	Description of trial design including type of trial (eg, parallel group, crossover, factorial, single group), allocation ratio, and framework (eg, superiority, equivalence, non-inferiority, exploratory)	5

For peer review only

**Methods: Participants, interventions, and outcomes**			
Study setting	#9	Description of study settings (eg, community clinic, academic hospital) and list of countries where data will be collected. Reference to where list of study sites can be obtained	5
Eligibility criteria	#10	Inclusion and exclusion criteria for participants. If applicable, eligibility criteria for study centres and individuals who will perform the interventions (eg, surgeons, psychotherapists)	5-6
Interventions: description	#11a	Interventions for each group with sufficient detail to allow replication, including how and when they will be administered	7
Interventions: modifications	#11b	Criteria for discontinuing or modifying allocated interventions for a given trial participant (eg, drug dose change in response to harms, participant request, or improving / worsening disease)	7
Interventions: adherance	#11c	Strategies to improve adherence to intervention protocols, and any procedures for monitoring adherence (eg, drug tablet return; laboratory tests)	7-8
Interventions:	#11d	Relevant concomitant care and interventions that are	7

For peer review only

concomitant care		permitted or prohibited during the trial	
Outcomes	#12	Primary, secondary, and other outcomes, including the specific measurement variable (eg, systolic blood pressure), analysis metric (eg, change from baseline, final value, time to event), method of aggregation (eg, median, proportion), and time point for each outcome. Explanation of the clinical relevance of chosen efficacy and harm outcomes is strongly recommended	8-9
Participant timeline	#13	Time schedule of enrolment, interventions (including any run-ins and washouts), assessments, and visits for participants. A schematic diagram is highly recommended (see Figure)	20
Sample size	#14	Estimated number of participants needed to achieve study objectives and how it was determined, including clinical and statistical assumptions supporting any sample size calculations	10
Recruitment	#15	Strategies for achieving adequate participant enrolment to reach target sample size	6
**Methods: Assignment of interventions (for controlled trials)**			
Allocation: sequence generation	#16a	Method of generating the allocation sequence (eg, computer-generated random numbers), and list of any factors for stratification. To reduce predictability of a	8

For peer review only

		random sequence, details of any planned restriction (eg, blocking) should be provided in a separate document that is unavailable to those who enrol participants or assign interventions	
Allocation concealment mechanism	#16b	Mechanism of implementing the allocation sequence (eg, central telephone; sequentially numbered, opaque, sealed envelopes), describing any steps to conceal the sequence until interventions are assigned	8
Allocation: implementation	#16c	Who will generate the allocation sequence, who will enrol participants, and who will assign participants to interventions	8
Blinding (masking)	#17a	Who will be blinded after assignment to interventions (eg, trial participants, care providers, outcome assessors, data analysts), and how	8
Blinding (masking): emergency unblinding	#17b	If blinded, circumstances under which unblinding is permissible, and procedure for revealing a participant's allocated intervention during the trial	8
**Methods: Data collection, management, and analysis**			
Data collection plan	#18a	Plans for assessment and collection of outcome, baseline, and other trial data, including any related processes to promote data quality (eg, duplicate measurements, training	10

For peer review only

		of assessors) and a description of study instruments (eg, questionnaires, laboratory tests) along with their reliability and validity, if known. Reference to where data collection forms can be found, if not in the protocol	
Data collection plan: retention	#18b	Plans to promote participant retention and complete follow-up, including list of any outcome data to be collected for participants who discontinue or deviate from intervention protocols	10-11
Data management	#19	Plans for data entry, coding, security, and storage, including any related processes to promote data quality (eg, double data entry; range checks for data values). Reference to where details of data management procedures can be found, if not in the protocol	10, 12
Statistics: outcomes	#20a	Statistical methods for analysing primary and secondary outcomes. Reference to where other details of the statistical analysis plan can be found, if not in the protocol	10-11
Statistics: additional analyses	#20b	Methods for any additional analyses (eg, subgroup and adjusted analyses)	11
Statistics: analysis population and missing data	#20c	Definition of analysis population relating to protocol non-adherence (eg, as randomised analysis), and any statistical methods to handle missing data (eg, multiple imputation)	11
**Methods: Monitoring**			
Data monitoring:	#21a	Composition of data monitoring committee (DMC);	11-12,

For peer review only

formal committee		summary of its role and reporting structure; statement of whether it is independent from the sponsor and competing interests; and reference to where further details about its charter can be found, if not in the protocol. Alternatively, an explanation of why a DMC is not needed	21-22
Data monitoring: interim analysis	#21b	Description of any interim analyses and stopping guidelines, including who will have access to these interim results and make the final decision to terminate the trial	12
Harms	#22	Plans for collecting, assessing, reporting, and managing solicited and spontaneously reported adverse events and other unintended effects of trial interventions or trial conduct	12
Auditing	#23	Frequency and procedures for auditing trial conduct, if any, and whether the process will be independent from investigators and the sponsor	12
**Ethics and dissemination**			
Research ethics approval	#24	Plans for seeking research ethics committee / institutional review board (REC / IRB) approval	12
Protocol amendments	#25	Plans for communicating important protocol modifications (eg, changes to eligibility criteria, outcomes, analyses) to relevant parties (eg, investigators, REC / IRBs, trial participants, trial registries, journals, regulators)	12-13

For peer review only

Consent or assent	#26a	Who will obtain informed consent or assent from potential trial participants or authorised surrogates, and how (see Item 32)	6
Consent or assent: ancillary studies	#26b	Additional consent provisions for collection and use of participant data and biological specimens in ancillary studies, if applicable	6
Confidentiality	#27	How personal information about potential and enrolled participants will be collected, shared, and maintained in order to protect confidentiality before, during, and after the trial	13
Declaration of interests	#28	Financial and other competing interests for principal investigators for the overall trial and each study site	2
Data access	#29	Statement of who will have access to the final trial dataset, and disclosure of contractual agreements that limit such access for investigators	10
Ancillary and post trial care	#30	Provisions, if any, for ancillary and post-trial care, and for compensation to those who suffer harm from trial participation	13
Dissemination policy: trial results	#31a	Plans for investigators and sponsor to communicate trial results to participants, healthcare professionals, the public, and other relevant groups (eg, via publication, reporting in results databases, or other data sharing arrangements), including any publication restrictions	13

For peer review only

Dissemination policy: authorship	#31b	Authorship eligibility guidelines and any intended use of professional writers	13
Dissemination policy: reproducible research	#31c	Plans, if any, for granting public access to the full protocol, participant-level dataset, and statistical code	10, 13

Appendices

Informed consent materials	#32	Model consent form and other related documentation given to participants and authorised surrogates	N/A
Biological specimens	#33	Plans for collection, laboratory evaluation, and storage of biological specimens for genetic or molecular analysis in the current trial and for future use in ancillary studies, if applicable	N/A

Notes:

- 21a: 11-12, 21-22 The SPIRIT Explanation and Elaboration paper is distributed under the terms of the Creative Commons Attribution License CC-BY-NC. This checklist was completed on 30. June 2021 using https://www.goodreports.org/, a tool made by the EQUATOR Network in collaboration with Penelope.ai

For peer review only
